# Supplementary material for: Influence of static disorder of charge transfer state on voltage loss in organic photovoltaics
Source: Nat Commun. 2021 Jun 15;12:3642. doi: 10.1038/s41467-021-23975-3 (PMC8206127; doi:10.1038/s41467-021-23975-3)
Supplement: Supplementary file 1 — Supplementary Information [file 41467_2021_23975_MOESM1_ESM.pdf]

Supplementary information

Influence of static disorder of charge transfer state on voltage loss in organic photovoltaics

Jun Yan[1,]*, Elham Rezasoltani[1], Mohammed Azzouzi[1], Flurin David Eisner[1], Jenny Nelson[1,]*

[1]Department of Physics and Centre for Processable Electronics, Imperial College London, SW7 2AZ, London, United Kingdom.

* Corresponding authors:

Jun Yan's email: j.yan17@imperial.ac.uk

Jenny Nelson's email: jenny.nelson@imperial.ac.uk

Supplementary Method 1. Experimental section

Solar cell fabrication. Inverted device structure is used in this study: ITO/ZnO/P3HT:NFA/MoOx/Ag, with ZnO and MoOx as the electron and hole transport layer, respectively. Pre-patterned ITO substrates were cleaned by acetone first, then detergent, deionized water, and isopropanol via sonication, and then dried by nitrogen gas gun before ZnO deposition. 30 nm ZnO thin films were then prepared via spinning coating ZnO precursor solution made from 219.5 mg zinc acetate dihydrate in 2ml 2-methoxyethanol with 60 ml mono-ethanolamine, followed by 150 °C thermal annealing for 10-15 mins. Active layers made from various D:A compositions (weight ratio from 20:80 to 80:20) were then deposited on top of ZnO film by spinning coating from 25 mg ml^{-1} solution in chlorobenzene, giving layers of 70±5 nm. Finally, 10 nm of MoOx followed by 100 nm of Ag layers were deposited on top of active layer by evaporation with a mask area of 0.045 cm^2.

Current-voltage (J-V). J-V characteristics were measured by a Keithley 2400 source-measurement unit under the AM 1.5G spectrum from a solar simulator (Oriel Instruments).

External quantum efficiency (EQE). The measurement was carried out using a grating spectrometer (CS260-RG-4-MT-D) to create monochromatic light combined with a tungsten halogen light source. The monochromatic light was chopped at ~200 Hz, and a Stanford Research Systems lock-in amplifier (SRS830 model) with an internal transimpedance amplifier of 10^6 V A^{-1} was used to detect the photocurrent. Long pass filters at 610, 715, 780, 850, and 1000 nm were used to filter out the scattered light from the monochromator. The spectra were taken from 300 to 1100 nm and calibrated by a silicon photodiode.

Electroluminescence (EL) and Photoluminescence (PL). EL measurement was carried out using a Shamrock 303 spectrograph combined with an iDUS InGaAs array detector cooled to -90 °C. The driving injection current is usually in the range of 5-50 mA. The obtained EL spectra intensity was calibrated with the spectrum from a calibrated halogen lamp. A 473nm diode laser was used as the excitation source for PL spectra, which were measured using the same spectrograph and detector system as for EL measurements at the open-circuit voltage of the devices.

Supplementary Method 2. Theoretical description of open circuit voltage

$V_{\rm oc}$ is determined by the difference between $V_{\rm oc,rad}$ and $\Delta V_{\rm nr}$ via[1–4],

$$V_{\rm oc} = V_{\rm oc,rad} - \Delta V_{\rm nr}. (1)$$

In Supplementary Equation (1), $V_{\rm oc,rad}$ can be computed by accounting for only radiative recombination losses using Shockley-Queisser theory[5] with an nonideal absorption profile[3], by

$$V_{\rm oc,rad} = \frac{k_B T}{q} \ln\left[\frac{\int_0^\infty {\rm EQE_{abs}}(\hbar\omega)\phi_S(\hbar\omega)\, d\hbar\omega}{\int_0^\infty {\rm EQE_{abs}}(\hbar\omega)\phi_{\rm BB}(\hbar\omega, T = 300K) d\hbar\omega} + 1\right], (2)$$

Where q is the elementary charge, and k_B is Boltzmann constant, T is temperature. ${\rm EQE_{abs}}(\hbar\omega)$ is the angular-independent external quantum efficiency for light absorption[4]. $\phi_S(\hbar\omega)$ is the standard AM 1.5G solar spectrum, and $\phi_{\rm BB}(\hbar\omega, T)$ is the black body radiation flux density per unit photon energy per second at temperature T, i.e. $\phi_{\rm BB}(\hbar\omega, T) = (\hbar\omega)^2/(4\pi^3\hbar^3c^2) \cdot \exp(-\hbar\omega/k_BT)$, where $\hbar\omega$ is the photon energy in eV; $\hbar$ is the reduced Planck constant in eV·s; ω is the angular frequency of the photon; c is the speed of the light. $\Delta V_{\rm nr}$ is determined by the external quantum efficiency of light emission (${\rm EQE_{EL}}$), via[2]

$$\Delta V_{\rm nr} = \frac{k_B T}{q} ln\left(\frac{1}{{\rm EQE_{EL}}}\right), (3)$$

where ${\rm EQE_{EL}}$ is related firstly to the rate constant of radiative (K_r) and nonradiative ($K_{\rm nr}$) decay of CT excitons, secondly to the outcoupling and optical properties of the cell through[1,6]

$${\rm EQE_{EL}} = \frac{p_e K_r}{K_{\rm nr} + p_e K_r}, (4)$$

with p_e being the emission probability[1] (see Supplementary Method 4 below for the expression).

Experimentally, $V_{\rm oc,rad}$ can be calculated by measuring ${\rm EQE_{abs}}(\hbar\omega)$ with a good sensitivity at CT absorption regime[3,7], and $\Delta V_{\rm nr}$ can be obtained either by subtracting $V_{\rm oc,rad}$ by $V_{\rm oc}$ [3] or by measuring ${\rm EQE_{EL}}$ [7]. Theoretically, we need to model ${\rm EQE_{abs}}(\hbar\omega)$, K_r and $K_{\rm nr}$[6], as discussed below.

Supplementary Method 3. Theoretical description of absorption and recombination from CT state with static disorder

To express the radiative recombination rate constant, we consider the rate of emission from the CT state to the ground state. We express the radiative rate constant using the operator ($O = \frac{e}{mc}\vec{\mathbf{A}}\cdot\vec{\mathbf{p}}$), $\vec{\mathbf{A}}$ is the vector potential of the electromagnetic field and $\vec{\mathbf{p}}$ the momentum operator[6]. With static disorder involved, using the Fermi's Golden (FG) rule and the dipole approximation, the emission rate constant (k_r) per unit photon energy ($\hbar\omega$) can be expressed using the transition dipole moment.[8] The absorption rate constant per photon energy (k_{abs}) can be expressed in a similar way as k_r except that we need to consider the photon density (W) in the volume. We consider the states in each CT state manifold (denoted as i in the subscript) share the same set of parameters. We assume quasi-thermal equilibrium (QTE) conditions, meaning that the occupation function of each electronic CT state should be considered in the expression for recombination, and that state occupation should follow Boltzmann statistics. Ultimately, when summing over all contributions from different CT manifolds (denoted as t), we have

$$k_{\mathrm{abs}}(\hbar\omega) = \frac{1}{Z_{\mathrm{abs}}}\sum_t \int_a^b \frac{W}{3\pi\epsilon_0\hbar^4}\left(\frac{\hbar\omega}{c}\right)^3 M_t(E_{\mathrm{CT}})^2 \mathrm{FCWD}_{\mathrm{abs},t}(\hbar\omega, E_{\mathrm{CT}}) c_t D_t(E_{\mathrm{CT}}) d(E_{\mathrm{CT}}), (5)$$

$$k_r(\hbar\omega) = \frac{1}{Z_{\mathrm{rec}}}\sum_t \int_a^b \frac{1}{3\pi\epsilon_0\hbar^4}\left(\frac{\hbar\omega}{c}\right)^3 M_t(E_{\mathrm{CT}})^2 \mathrm{FCWD}_{\mathrm{rec},t}(\hbar\omega, E_{\mathrm{CT}}) c_t D_t(E_{\mathrm{CT}}) \exp\left(-\frac{E_{\mathrm{CT}}}{k_B T}\right) d(E_{\mathrm{CT}}), (6)$$

Where W is the photon density and accounts for the strength of electro-magnetic field around the molecule;[9] ϵ_0 is the permittivity of the free space; t is the order of CT state manifold; $M_t(E_{\mathrm{CT}})$ is the transition dipole moment for CT manifold t and is related to the oscillator strength of the CT manifold ($f_{\mathrm{osc},t}$) under the dipole approximation[10,11], via $M_t(E_{\mathrm{CT}}) = \sqrt{(3/2)q^2\hbar^2 f_{\mathrm{osc},t}/(E_{\mathrm{CT}} m_e)}$, with m_e the electron mass. $\mathrm{FCWD}_{\mathrm{abs},t\ \mathrm{or\ rec},t}(\hbar\omega, E_{\mathrm{CT}})$ is the Franck-Condon weighted density of state for absorption or emission at $\hbar\omega$ for a certain CT manifold t, and it sums all the wavefunction overlaps between the vibrational modes of initial and final state, as follow

$$\mathrm{FCWD}_{\mathrm{abs},t}(\hbar\omega, E_{\mathrm{CT}}) = \frac{1}{\sqrt{4\pi\lambda_{o,t}k_BT}} \times \sum_{m=0}^{\infty}\sum_{n=0}^{\infty}\frac{e^{-S_t}S_t^{n-m}m!}{n!}\left[(L_m^{n-m}(S_t)\right]^2 \exp\left[-\frac{\left(-\hbar\omega + E_{\mathrm{CT}} + \lambda_{o,t} + (n-m)\hbar\Omega_t\right)^2}{4\lambda_{o,t}k_BT}\right]\exp\left(-\frac{m\hbar\Omega_t}{k_BT}\right), \quad (7)$$

$$\mathrm{FCWD}_{\mathrm{rec},t}(\hbar\omega, E_{CT}) = \frac{1}{\sqrt{4\pi\lambda_{o,t}k_BT}} \times \sum_{m=0}^{\infty}\sum_{n=0}^{\infty}\frac{e^{-S_t}S_t^{n-m}m!}{n!}\left[(L_m^{n-m}(S_t)\right]^2 \exp\left[-\frac{\left(\hbar\omega - E_{\mathrm{CT}} + \lambda_{o,t} + (n-m)\hbar\Omega_t\right)^2}{4\lambda_{o,t}k_BT}\right]\exp\left(-\frac{m\hbar\Omega_t}{k_BT}\right), \quad (8)$$

where $\lambda_{o,t}$ and $\lambda_{i,t}$ are the low frequency and high frequency reorganization energy for CT manifold t, respectively, $S_t = \lambda_{i,t}/\hbar\Omega_t$ is the Huang Rhys factor[12], $\hbar\Omega_t$ is the averaged harmonic energy spacing, typically 0.15-0.20 eV for molecules made of many carbon-carbon bonds[6,13] and m and n are the quantum numbers of the vibrational mode of the initial and final state. respectively. $L_m^{n-m}(S_t)$ is then the generalized Laguerre polynomial of degree m[6]. The factor Z_{abs} and Z_{rec} in Supplementary Equation (5) and Supplementary Equation (6) are the partition functions, and are defined as the sum of occupation of vibrational CT states for absorption, and as the sum of occupation of both vibrational and electronic CT states for recombination, and are given by

$$Z_{\mathrm{abs}} = \sum_t\sum_{m=0}^{\infty}\exp\left(-\frac{m\hbar\Omega_t}{k_BT}\right), (9)$$

$$Z_{\mathrm{rec}} = \sum_t\left\{\sum_{m=0}^{\infty}\exp\left(-\frac{m\hbar\Omega_t}{k_BT}\right)\int_a^b c_tD_t(E_{\mathrm{CT}})\exp\left(-\frac{E_{\mathrm{CT}}}{k_BT}\right)d(E_{\mathrm{CT}})\right\}. (10)$$

K_r (s^{-1}) is obtained by integrating Supplementary Equation (6) over $\hbar\omega$, and K_{nr} (s^{-1}) can be expressed in a similar fashion as k_r (see Supplementary Equation (6)) using Fermi's Golden (FG) rule[14] and BO approximation[15] as a product of the electronic coupling V between the CT state and ground state and the Franck-Condon weighted density of states. And we have

$$K_r = \int_0^{\infty} k_r(\hbar\omega) d\hbar\omega\,, (11)$$

$$K_{\mathrm{nr}} = \frac{1}{Z_{\mathrm{rec}}} \sum_t \int_a^b \frac{2\pi}{\hbar} V_t(E_{\mathrm{CT}})^2 \mathrm{FCWD}_{\mathrm{rec},t}(0, E_{\mathrm{CT}}) c_t D_t(E_{\mathrm{CT}}) \exp\left(-\frac{E_{\mathrm{CT}}}{k_B T}\right) d(E_{\mathrm{CT}})\,, (12)$$

where $V_t(E_{\mathrm{CT}})$ is the electronic coupling (EC) between CT and ground state described by generalized Mulliken-Hush method[16,17]. $\mathrm{FCWD}_{\mathrm{rec},t}(0, E_{\mathrm{CT}})$ follows Supplementary Equation (8) with $\hbar\omega = 0$.

We have now the rate constants K_r and K_{nr} to model ΔV_{nr} using Supplementary Equation (3). To predict V_{oc}, what we also need is $V_{\mathrm{oc,rad}}$, which is calculated using $\mathrm{EQE}_{\mathrm{abs}}(\hbar\omega)$ in Supplementary Equation (2).

Supplementary Method 4. Absorptance and emission probability

Knowing the absorption rate constant (k_{abs}) as a function of photon energy ($\hbar\omega$), the absorption coefficient ($\alpha_{\text{abs}}(\hbar\omega)$) can be calculated using Einstein coefficient as[6,14,17]

$$\alpha_{\text{abs}}(\hbar\omega) = \frac{n\pi\hbar^3 c^2}{2W\mathcal{V}} \frac{1}{(\hbar\omega)^2} k_{\text{abs}}(\hbar\omega), (13)$$

Where n is the refractive index, and $\mathcal{V}$ is the volume, $\mathcal{V}_{\text{CT}} = \mathcal{V}_E N_{\text{CT/LE}}$, with $\mathcal{V}_E = 1\ \text{Å}^3$. $N_{\text{CT/LE}}$ is the volume density ratio between CT and excitonic state. The total absorption coefficient $\alpha_{\text{abs}}^{\text{total}}(\hbar\omega)$ is then a superposition of the absorption from CT state ($\alpha_{\text{abs}}^{\text{CT}}$) and excitonic state ($\alpha_{\text{abs}}^{\text{LE}}$) calculated by[1,6]

$$\alpha_{\text{abs}}^{\text{total}}(\hbar\omega) = \begin{cases} \alpha_{\text{abs}}^{\text{CT}} + \alpha_{\text{abs}}^{\text{LE}}, & for\ 0 < \hbar\omega < E_{\text{LE}} + 2k_B T \\ \alpha_0 \times \sqrt{\dfrac{\hbar\omega - E_{\text{LE}}}{k_B T}}, & for\ E_{\text{LE}} + 2k_B T < \hbar\omega \end{cases}. (14)$$

$\alpha_{\text{abs}}^{\text{LE}}$ can be modelled using Supplementary Equation (5) and Supplementary Equation (13) with E_{CT} replaced by E_{LE}. Note that we can also consider a density distribution of excitonic states, however, for the interest of this work, we consider excitonic state has a single energy sitting at E_{LE}. We consider that for energies above $E_{\text{LE}} + 2k_B T$, $\alpha_{\text{abs}}^{\text{total}}$ follows the square root law of a direct semiconductor[1], with $\alpha_0 = 2/d$, where d is the thickness of the device.

Using Beer-Lambert law without interference, zero reflectance at the surface and unity reflectance from the back electrode, the absorptance ($A(\hbar\omega)$) of the device can be expressed as follow[1]

$$A(\hbar\omega) = 1 - \exp\left[-2d\alpha_{\text{abs}}^{\text{total}}(\hbar\omega)\right], (15)$$

Assuming perfect electron transport and no losses of photoinduced charges, we can consider the absorptance $A(\hbar\omega)$ as the ideal $\text{EQE}_{\text{abs}}(\hbar\omega)$ of the device. We finally calculate the emission probability p_{e} using[1]

$$p_e = \frac{1}{4n^2 d} \frac{\int_0^\infty A(\hbar\omega)\phi_{\text{BB}}(\hbar\omega) d\hbar\omega}{\int_0^\infty \alpha_{\text{abs}}^{\text{total}}(\hbar\omega)\phi_{\text{BB}}(\hbar\omega) d\hbar\omega}. (16)$$

Supplementary Method 5. The principle of detailed balance

Following the derivation by Rau, to obey the principle of detailed balance, the following equation should be validated[2]:

$$A(\hbar\omega) \propto \frac{k_r(\hbar\omega)}{\phi_{\mathrm{BB}}(\hbar\omega)}. (17)$$

Supplementary Equation (17) correlates the emission ($k_r(\hbar\omega)$) to absorptance ($A(\hbar\omega)$). Another way of examining detailed balance is to utilize the absorption coefficient of CT states ($\alpha_{\mathrm{abs}}^{\mathrm{CT}}(\hbar\omega)$), which is more convenient using our model. Here, we rewrite Supplementary Equation (15) using a Taylor expansion as:

$$\alpha_{\mathrm{abs}}^{\mathrm{CT}}(\hbar\omega) = -\frac{1}{2d}\ln(1-A) = -\frac{1}{2d}\left(-A-\frac{A^2}{2}-\frac{A^3}{3}-\frac{A^4}{4}-\cdots\right), (18)$$

In the case of absorptance from CT states, A is much smaller than 1. Therefore, in order to obey detailed balance, the proportionality shown below should apply:

$$\alpha_{\mathrm{abs}}^{\mathrm{CT}}(\hbar\omega) \approx \frac{A(\hbar\omega)}{2d} \propto A(\hbar\omega) \propto \frac{k_r(\hbar\omega)}{\phi_{\mathrm{BB}}(\hbar\omega)}, (19)$$

Therefore, both Supplementary Equation (17) and Supplementary Equation (19) can be used to test the principle of detailed balance. Practically, $A(\hbar\omega)$ is easier to measure, therefore, Supplementary Equation (17) is the most frequently used method in experiments assuming the device is not limited by charge collection, i.e. $A(\hbar\omega)\sim\mathrm{EQE}(\hbar\omega)$. However, in the simulations, we find that Supplementary Equation (19) offers a better way to test the principle of detailed balance, as we can expand the fitting range to the full photon energy spectrum (examples can be seen in Supplementary Fig. 2).

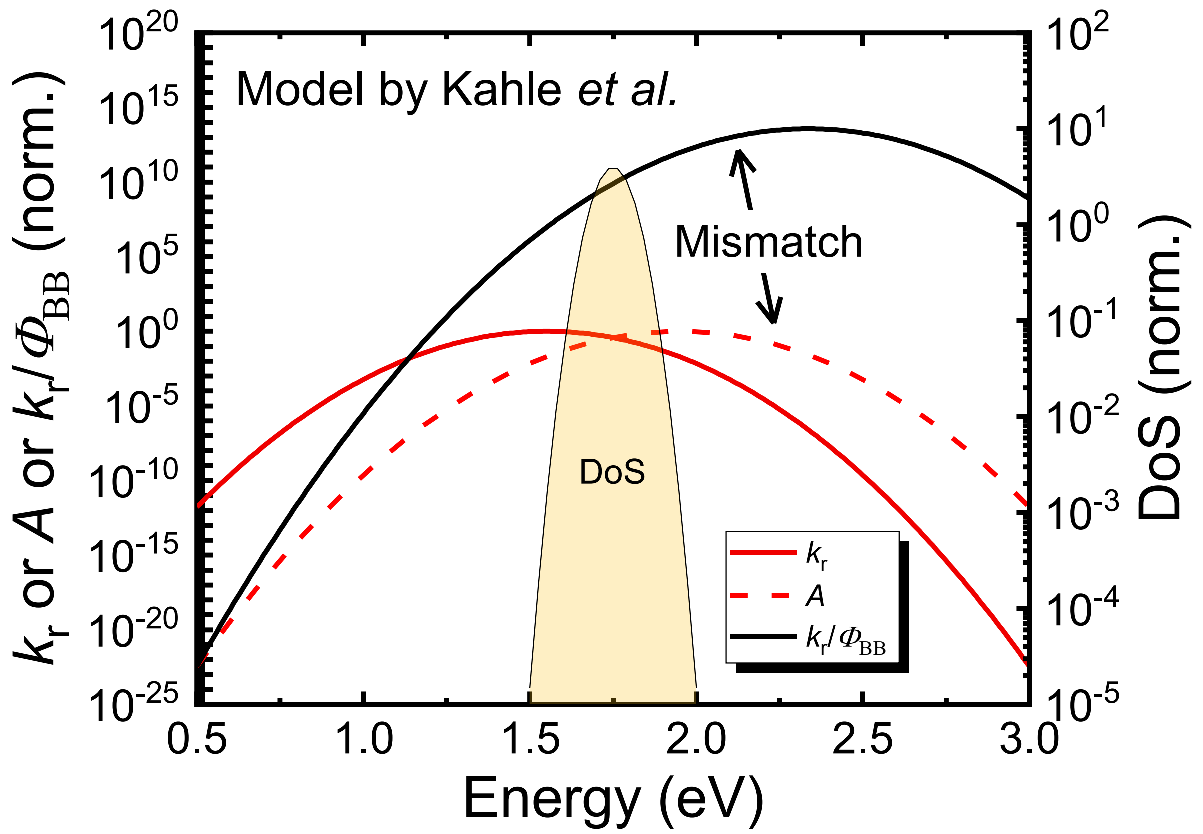

Supplementary Fig. 1. Examination of detailed balance using model by Kahle *et al.*[18]. Normalized rate constant per photon energy of emission (k_r), absorptance (A), and $k_r/\phi_{\rm BB}$ are calculated and shown. A clear mismatch between normalized A and k_r/ϕ_{BB} is seen in the model results by Kahle *et al.* [18] We also note here that other models in Ref. [19–21] are essentially based on model by Kahle *et al.* [18], therefore, only the result of Kahle model is shown. Please see Supplementary Note 1 for the details for the simulation.

Supplementary Note 1. Equations for modelling Supplementary Fig. 1

The equations used to perform the simulation in Supplementary Fig. 1 are the high temperature limit of Equation (8a-b) in Ref. [18] (i.e. Kahle model), which is also quoted as Equation (9) and Equation (10) in Ref. [19], as shown here:

$$rk_r(\hbar\omega) = \frac{k_r(\hbar\omega)}{\hbar\omega} \propto \exp\left\{-\frac{[\hbar\omega - (E_{\mathrm{CT}} - \lambda_o)]^2}{4\lambda_o k_B T + 2\sigma^2}\right\}, (20)$$

$$rA(\hbar\omega) = A(\hbar\omega) \cdot \hbar\omega \propto \exp\left\{-\frac{[\hbar\omega - (E_{\mathrm{CT}} + \lambda_o)]^2}{4\lambda_o k_B T + 2\sigma^2}\right\}, (21)$$

Where $rk_r(\hbar\omega)$ and $rA(\hbar\omega)$ are the reduced rate constant of emission and reduced absorptance. Note here we use the same notation for emission and absorptance as in our model to avoid confusion. In the simulation, the parameters were chosen as: $E_{\mathrm{CT}} = 1.75\ eV$, $\lambda_o = 0.2\ eV$, and $\sigma = 0.1\ eV$.

Here, we also use an analytical method to check the validation of detailed balance of the Kahle model. In order to obey detailed balance, Supplementary Equation (17) must be valid. To make it clearer analytically, we here get rid of the energy term ($\hbar\omega$) except for the exponential term in Supplementary Equation (17), then we have:

$$\frac{rk_r(\hbar\omega)}{rA(\hbar\omega)} \propto \exp\left(-\frac{\hbar\omega}{k_B T}\right), (22)$$

However, when we perform the division using Supplementary Equation (20) and Supplementary Equation (21) we have:

$$\frac{rk_r(\hbar\omega)}{rA(\hbar\omega)} \propto \frac{\exp\left\{-\frac{[\hbar\omega - (E_{\mathrm{CT}} - \lambda_o)]^2}{4\lambda_o k_B T + 2\sigma^2}\right\}}{\exp\left\{-\frac{[\hbar\omega - (E_{\mathrm{CT}} + \lambda_o)]^2}{4\lambda_o k_B T + 2\sigma^2}\right\}} = \exp\left\{-\frac{4(\hbar\omega - E_{\mathrm{CT}})\lambda_o}{4\lambda_o k_B T + 2\sigma^2}\right\}$$

$$\propto \exp\left\{-\frac{4\hbar\omega\lambda_o}{4\lambda_o k_B T + 2\sigma^2}\right\} \neq \exp\left(-\frac{\hbar\omega}{k_B T}\right), (23)$$

Therefore, the principle of detailed balance cannot be satisfied using Supplementary Equation (20) and Supplementary Equation (21) when σ is non-zero.

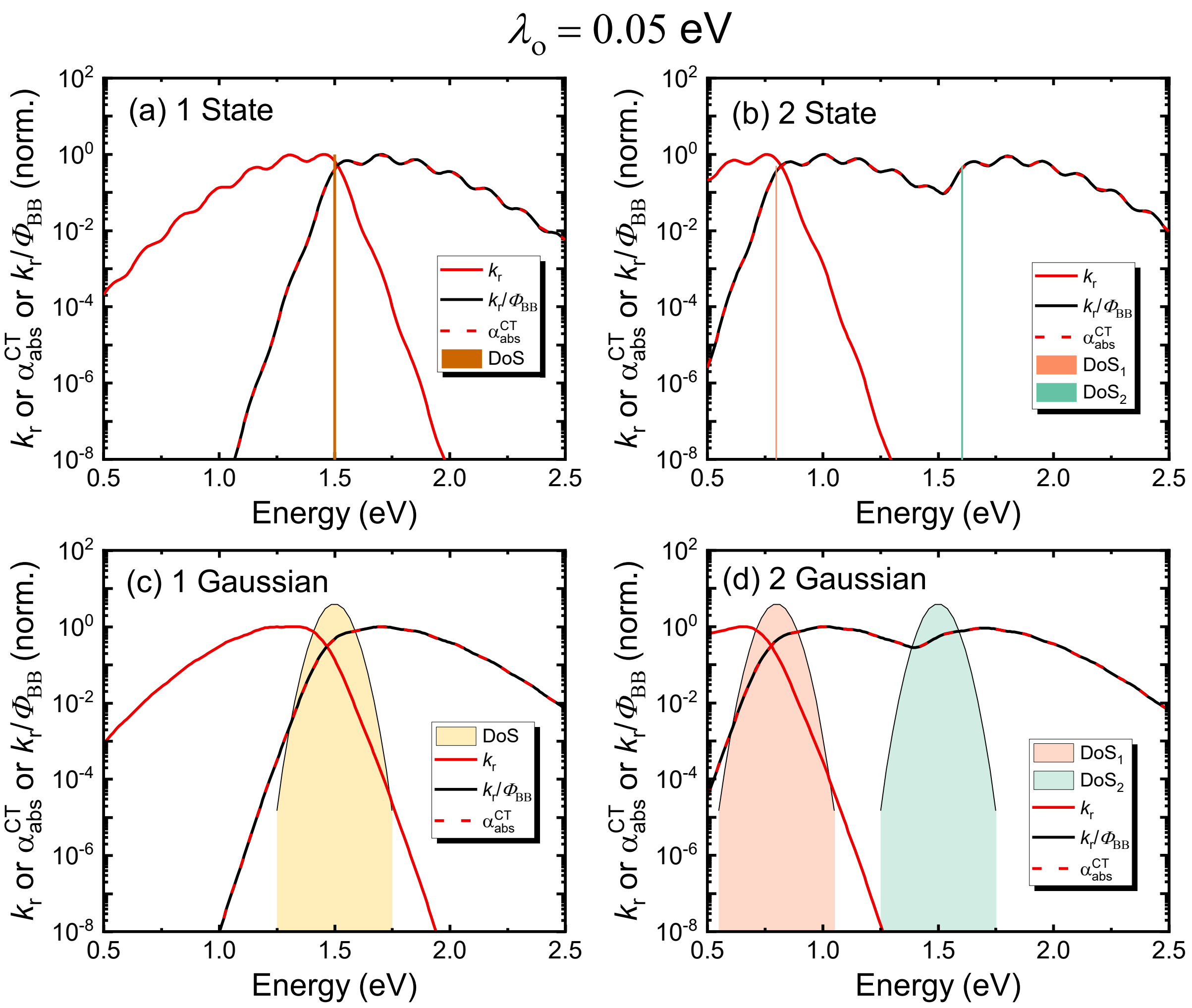

Supplementary Fig. 2. Examination of detailed balance with various $g(E_{\mathrm{CT}})$ shapes using our model. (a) Normalized rate constant per photon energy of emission (k_r), absorption coefficient of CT states ($\alpha_{\mathrm{abs}}^{\mathrm{CT}}$), and k_r/ϕ_{BB} for $g(E_{\mathrm{CT}})$ with (a) 1 state; (b) 2 states; (c) 1 gaussian; (d) 2 gaussians. We set $\lambda_o = 0.05$ eV to visualize the vibronic peaks. Each gaussian DoS has a σ of 0.05 eV and contains 20 states. The rest parameters follow Supplementary Table 1.

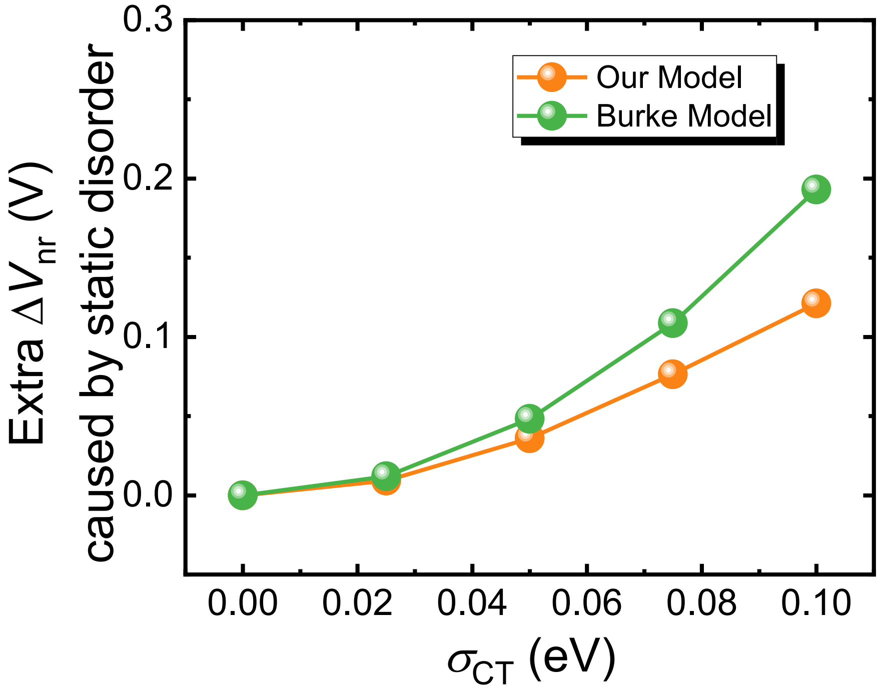

Supplementary Fig. 3. Extra nonradiative voltage losses caused by static disorder using Burke model[22] **as compared to the results from our model.** In both models, we use one gaussian density of state of CT state. We note that energy gap law is not considered in Burke model, and the extra nonradiative voltage loss follows $\sigma_{\mathrm{CT}}^2/2k_BT$. We see that Burke model significantly overestimates the extra nonradiative voltage losses as compared to our mode results. The parameters used to calculate the nonradiative voltage losses using our model are listed in Supplementary Table 1.

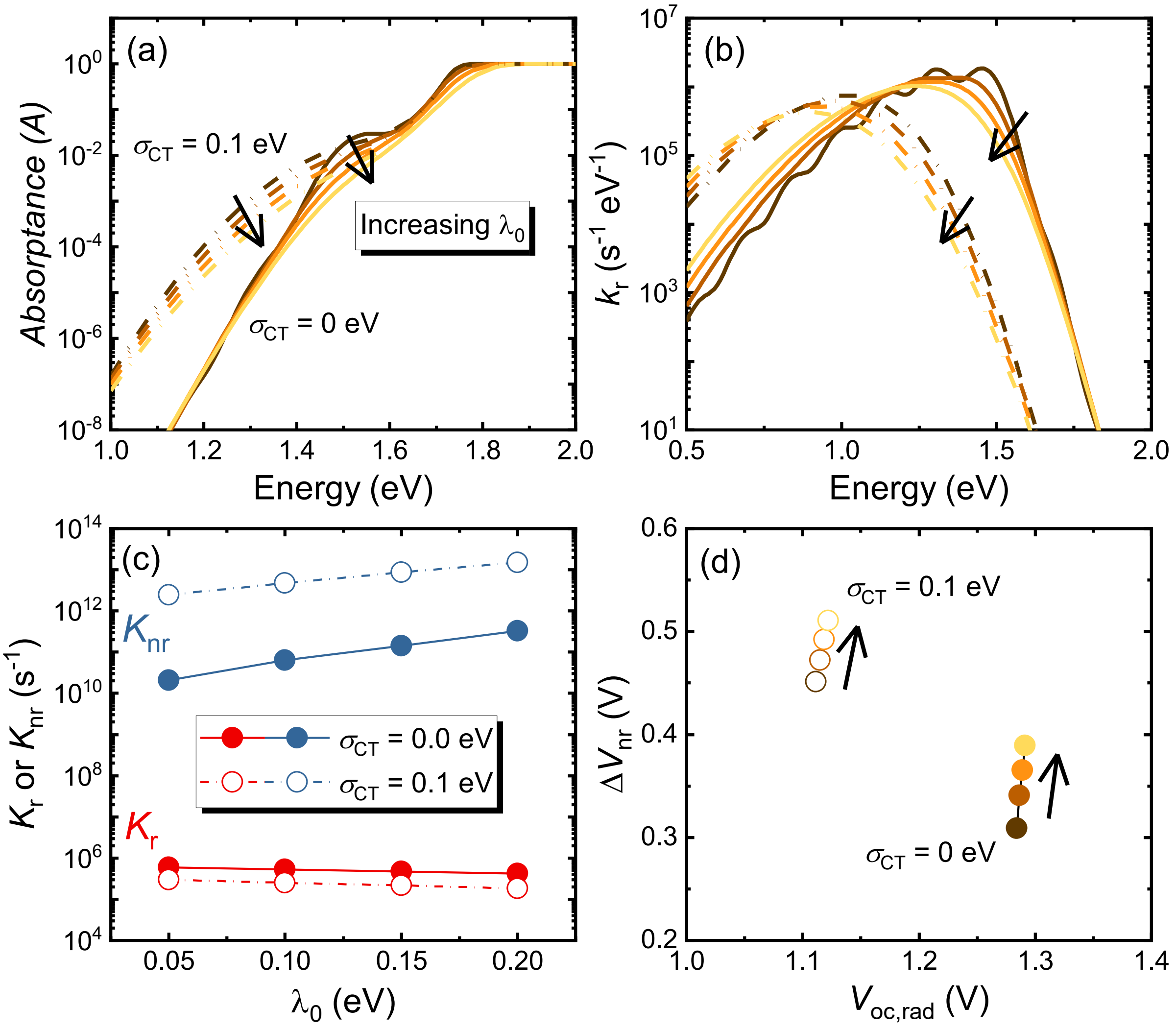

Supplementary Fig. 4. Effect of λ_o on (a) absorption spectrum, (b) emission spectrum, (c) rate constants, and (d) voltage loss plot with varied Gaussian width of $g(E_{CT})$ (1 Gaussian). Calculations are done with two different static disorder values in a simple 1 gaussian DoS, i.e. 0.0 eV and 0.1 eV. Solid line and dash-dotted line represent the case where $\sigma_{CT} = 0.0\ eV$ and the case where $\sigma_{CT} = 0.1\ eV$, respectively. The arrow indicates the direction of increasing variable values. The parameters used to produce the figures here are listed in Supplementary Table 4.

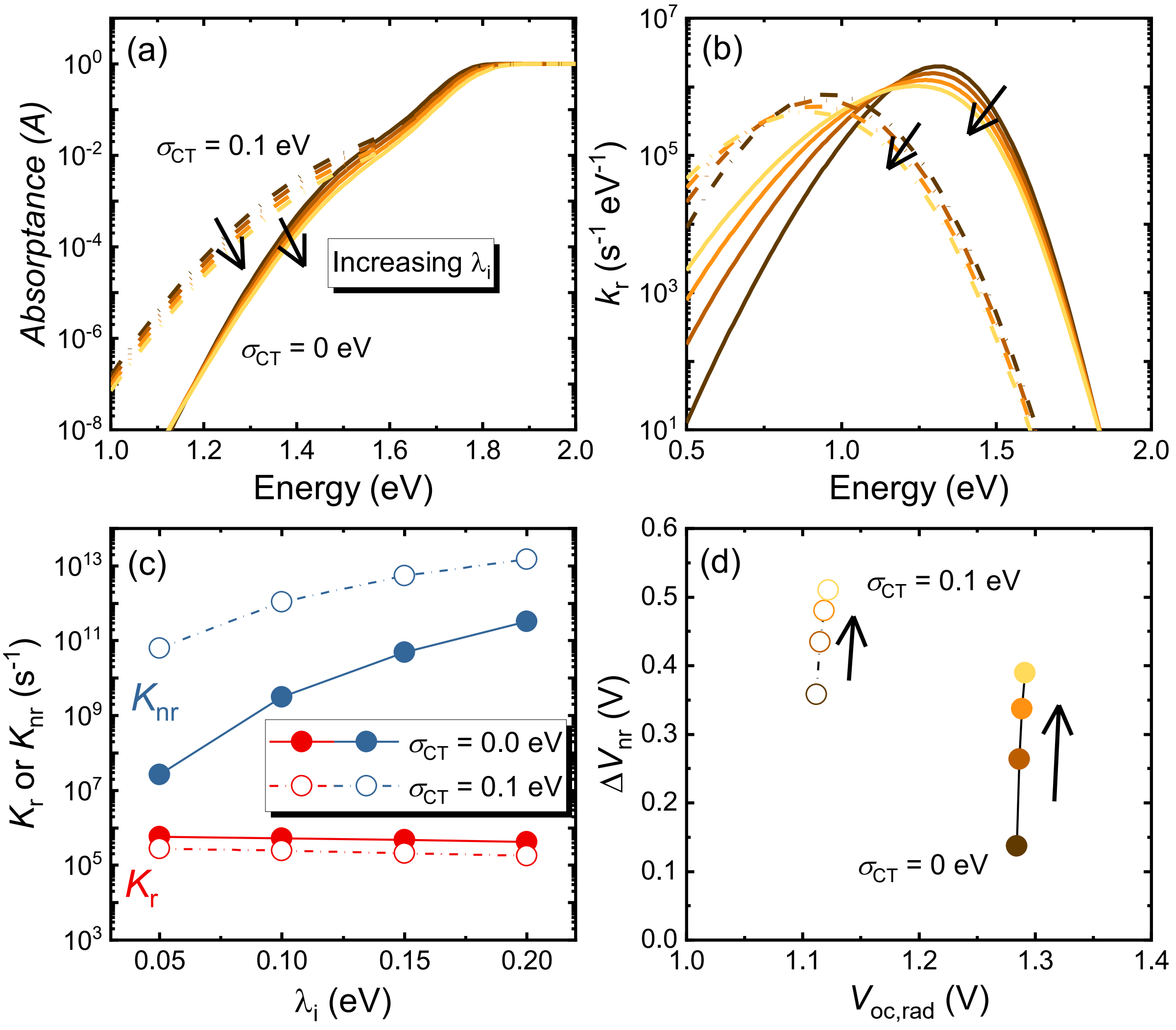

Supplementary Fig. 5. Effect of λ_i on (a) absorption spectrum, (b) emission spectrum, (c) rate constants, and (d) voltage loss plot with varied Gaussian width of $g(E_{CT})$ (1 Gaussian). Calculations are done with two different static disorder values in a simple 1 gaussian DoS, i.e. 0.0 eV and 0.1 eV. Solid line and dash-dotted line represent the case where $\sigma_{CT} = 0.0\ eV$ and the case where $\sigma_{CT} = 0.1\ eV$, respectively. The arrow indicates the direction of increasing variable values. The parameters used to produce the figures here are listed in Supplementary Table 4.

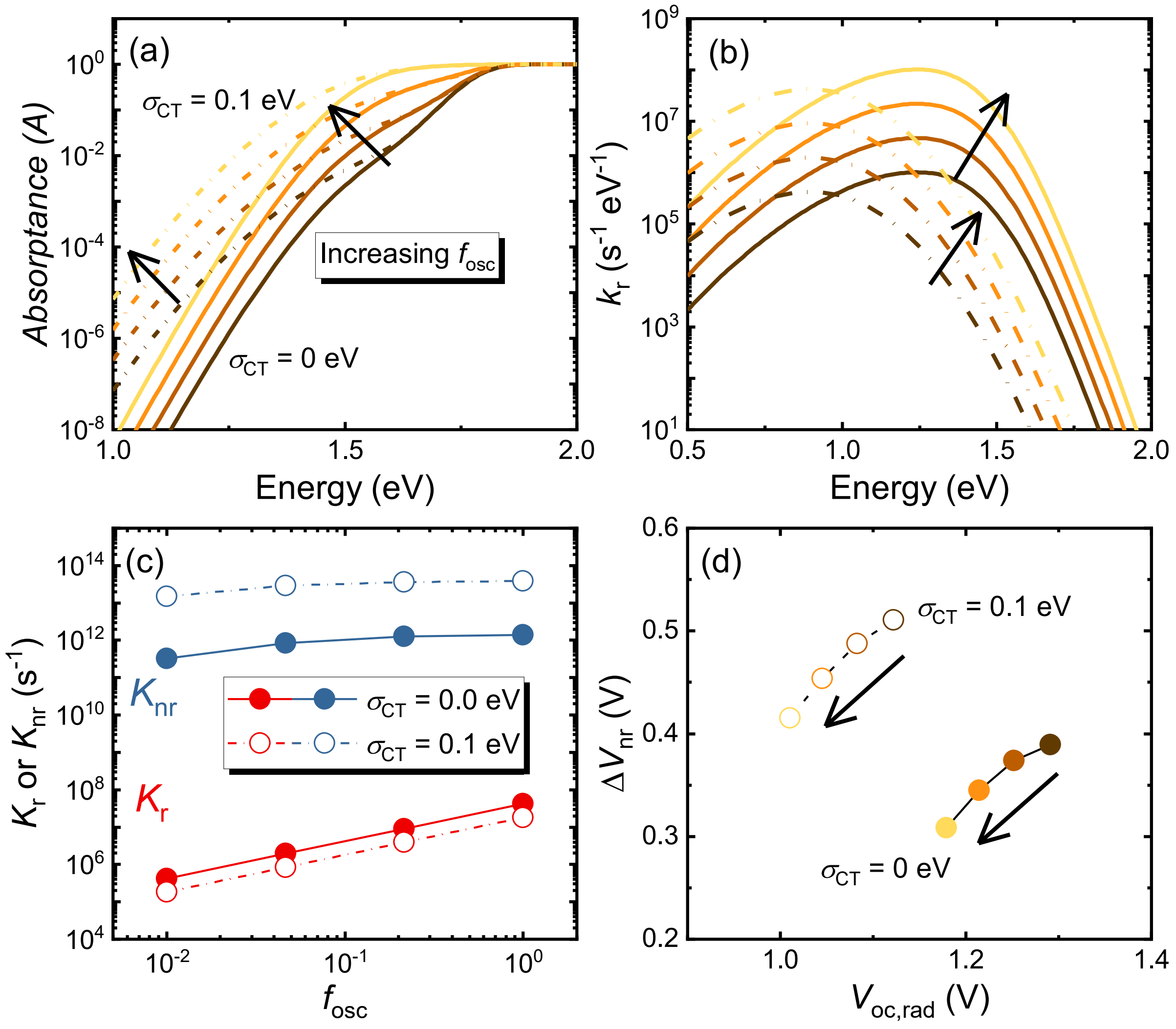

Supplementary Fig. 6. Effect of f_{osc} on (a) absorption spectrum, (b) emission spectrum, (c) rate constants, and (d) voltage loss plot with varied Gaussian width of $g(E_{CT})$ (1 Gaussian). Calculations are done with two different static disorder values in a simple 1 gaussian DoS, i.e. 0.0 eV and 0.1 eV. Solid line and dash-dotted line represent the case where $\sigma_{CT} = 0.0\ eV$ and the case where $\sigma_{CT} = 0.1\ eV$, respectively. The arrow indicates the direction of increasing variable values. The parameters used to produce the figures here are listed in Supplementary Table 4.

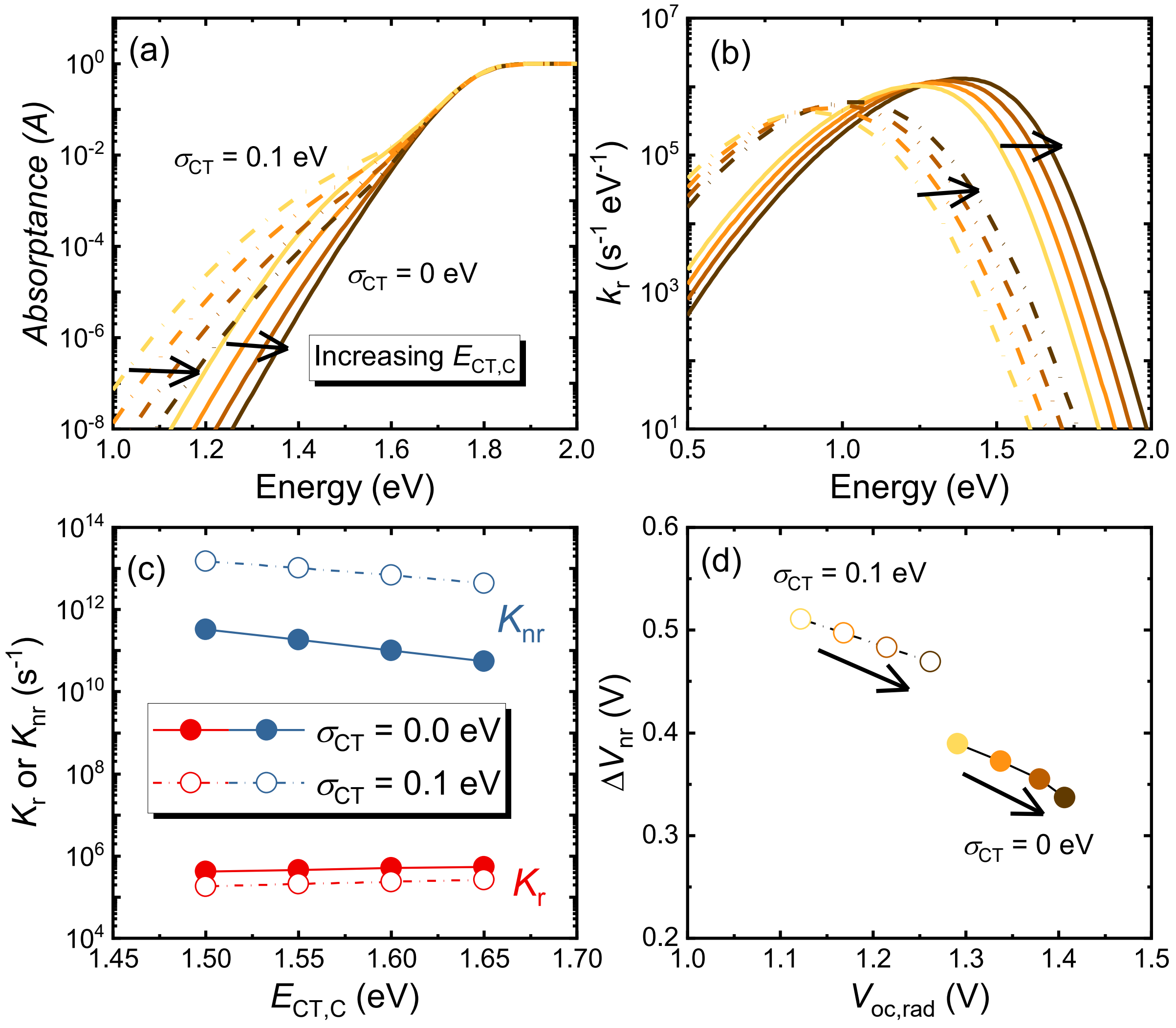

Supplementary Fig. 7. Effect of $E_{CT,C}$ on (a) absorption spectrum, (b) emission spectrum, (c) rate constants, and (d) voltage loss plot with varied Gaussian width of $g(E_{CT})$ (1 Gaussian). Calculations are done with two different static disorder values in a simple 1 gaussian DoS, i.e. 0.0 eV and 0.1 eV. Solid line and dash-dotted line represent the case where $\sigma_{CT} = 0.0\ eV$ and the case where $\sigma_{CT} = 0.1\ eV$, respectively. The arrow indicates the direction of increasing variable values. The parameters used to produce the figures here are listed in Supplementary Table 4.

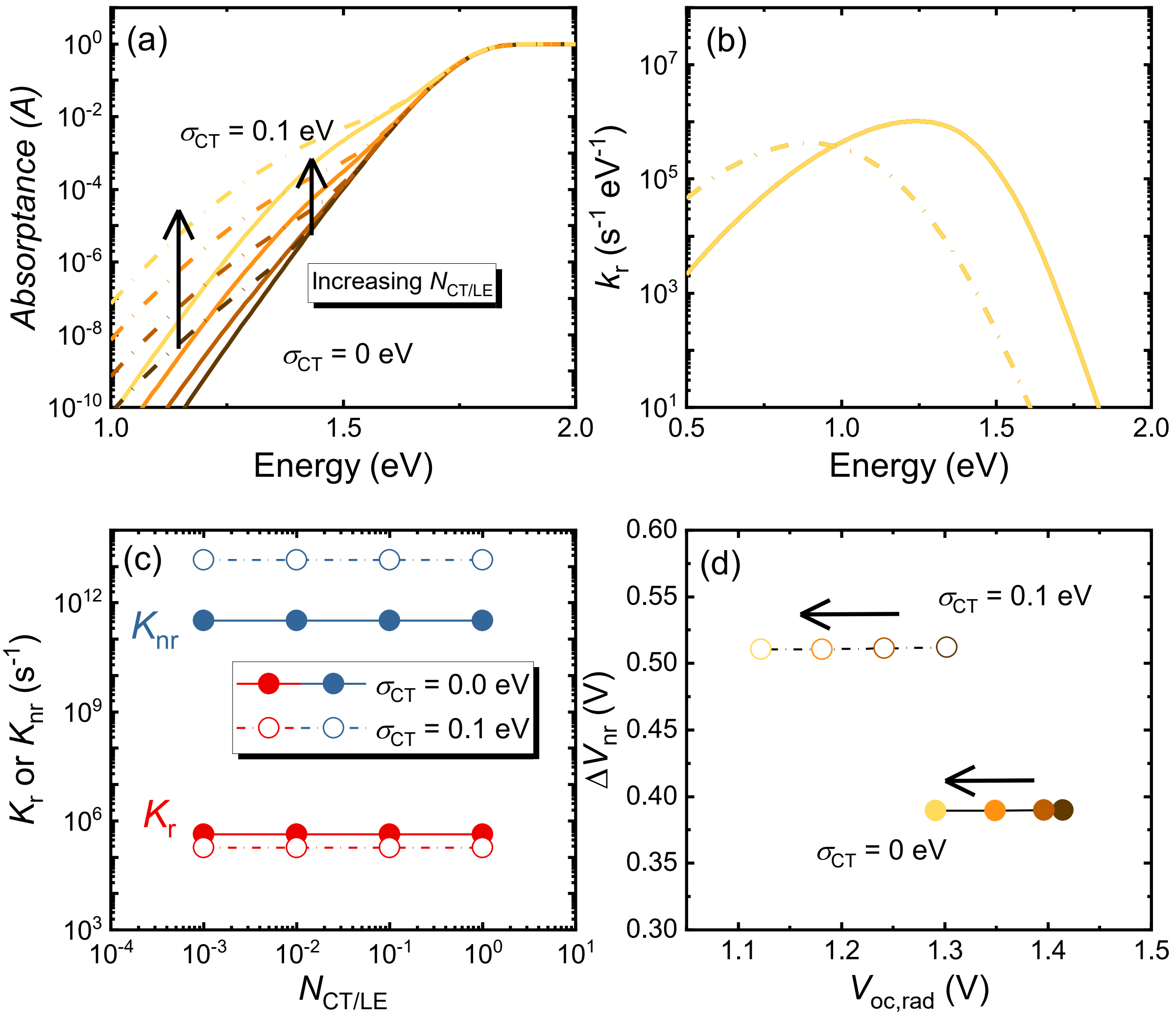

Supplementary Fig. 8. Effect of $N_{CT/LE}$ on (a) absorption spectrum, (b) emission spectrum, (c) rate constants, and (d) voltage loss plot with varied Gaussian width of $g(E_{CT})$ (1 Gaussian). Calculations are done with two different static disorder values in a simple 1 gaussian DoS, i.e. 0.0 eV and 0.1 eV. Solid line and dash-dotted line represent the case where $\sigma_{CT} = 0.0\ eV$ and the case where $\sigma_{CT} = 0.1\ eV$, respectively. The arrow indicates the direction of increasing variable values. The parameters used to produce the figures here are listed in Supplementary Table 4.

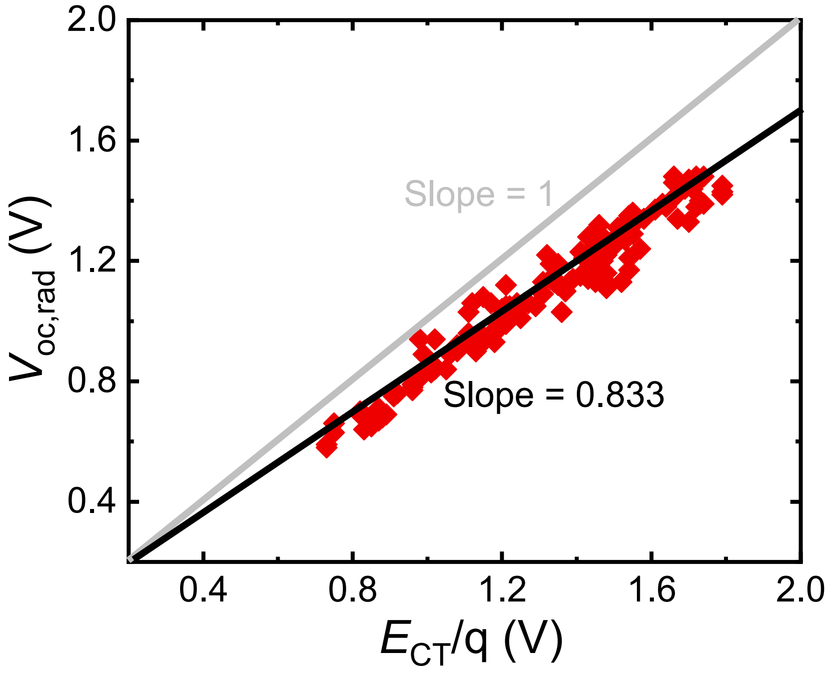

Supplementary Fig. 9. Experimental $V_{oc,rad}$ as a function of E_{CT}. A linear correlation between $V_{oc,rad}$ and E_{CT} is seen, and can roughly be expressed as $V_{oc,rad} = 0.833E_{CT}/q$. Data are taken from Ref. [23].

Supplementary Note 2. Voltage and efficiency limit (1 gaussian)

V_{oc} and PCE limits with varied absorber optical gap (E_g) and σ_{CT} of 1 gaussian $g(E_{\mathrm{CT}})$ have been calculated following the method introduced previously[6] and shown in Supplementary Fig. 10. The parameters chosen here for simulating those limits are listed in Supplementary Table 5. We consider that the driving force is zero for the simulations, i.e. $E_{\mathrm{CT}} = E_g$, based on the fact that efficient OPVs with zero driving force have been reported[24,25]. Supplementary Fig. 10a shows the calculated $V_{\mathrm{oc,rad}}$ and $V_{\mathrm{oc,nr}}$ as a function of E_g/q. Without static disorder, i.e. $\sigma_{\mathrm{CT}} = 0\ eV$, $V_{\mathrm{oc,rad}}$ scales linearly with E_g/q with a slope smaller than 1, while $V_{\mathrm{oc,nr}}$ increases faster than $V_{\mathrm{oc,rad}}$ with E_g/q (slope > 1), consistent with previous findings[6,13]. With 0.1 eV of σ_{CT}, we see a notable drop of both $V_{\mathrm{oc,rad}}$ and $V_{\mathrm{oc,nr}}$, however energy gap law on ΔV_{nr} is still obeyed[13]. As a result, both radiative ($\mathrm{PCE_{rad}}$) and nonradiative ($\mathrm{PCE_{nr}}$) efficiency limit are reduced significantly, as shown in Supplementary Fig. 10b.

To have an idea of how V_{oc} and PCE limits would scale with σ_{CT}, we carry out further calculations for the devices with the optimum band gap ($E_g = 1.4$ eV) as derived from Shockley-Queisser (SQ) limit[5], which is also close to E_g of the best OPV[26]. Without static disorder, $V_{\mathrm{oc,rad}}$ and $V_{\mathrm{oc,nr}}$ are 1.05 and 0.9 V giving an $\Delta V_{\mathrm{nr}} = \mathbf{0.15}$ eV, and $\mathrm{PCE_{rad}}$ and $\mathrm{PCE_{nr}}$ are 33% and 28%, respectively. Upon introducing a finite σ_{CT}, we observe a notable decline of both limits for V_{oc}, as shown in Supplementary Fig. 10c. As a result, both $\mathrm{PCE_{rad}}$ and $\mathrm{PCE_{nr}}$ have been reduced notably due to extra radiative and nonradiative losses induced by static disorder in Supplementary Fig. 10d. With 0.1 eV of σ_{CT}, we observe a 33% reduction of both $V_{\mathrm{oc,nr}}$ and $\mathrm{PCE_{nr}}$. These modelling results suggest that minimizing σ_{CT} is crucial for maintaining high voltage and efficiency.

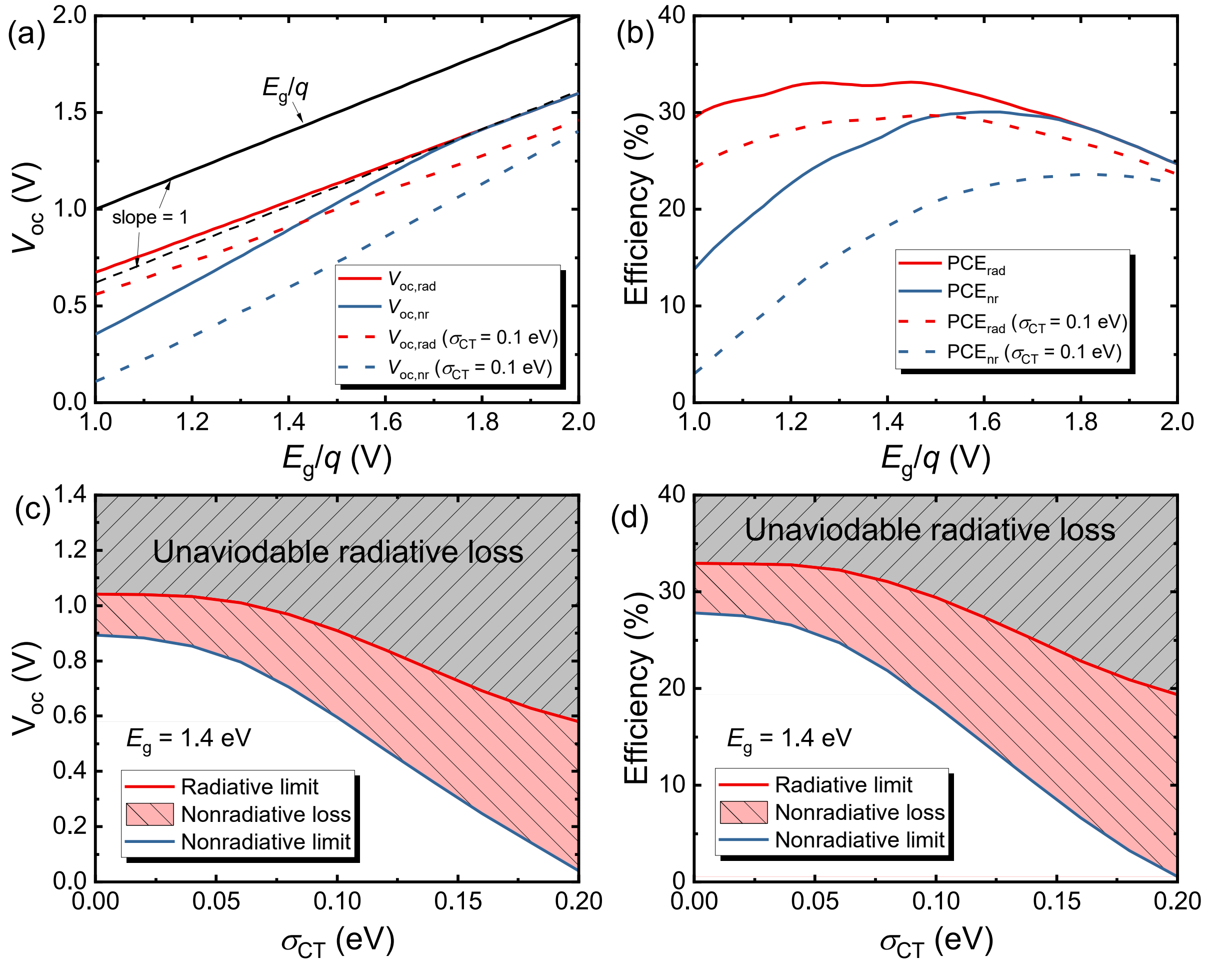

Supplementary Fig. 10. Simulated theoretical limit in the presence of $g(E_{CT})$. (a) V_{oc} and (b) PCE as a function of optical gap (E_g); (c) V_{oc} and (d) PCE as a function of σ_{CT}, with E_g chosen to be 1.4 eV. Unavoidable radiative losses are displayed as grey shaded area. We set $E_{CT} = E_g$ in all plots.

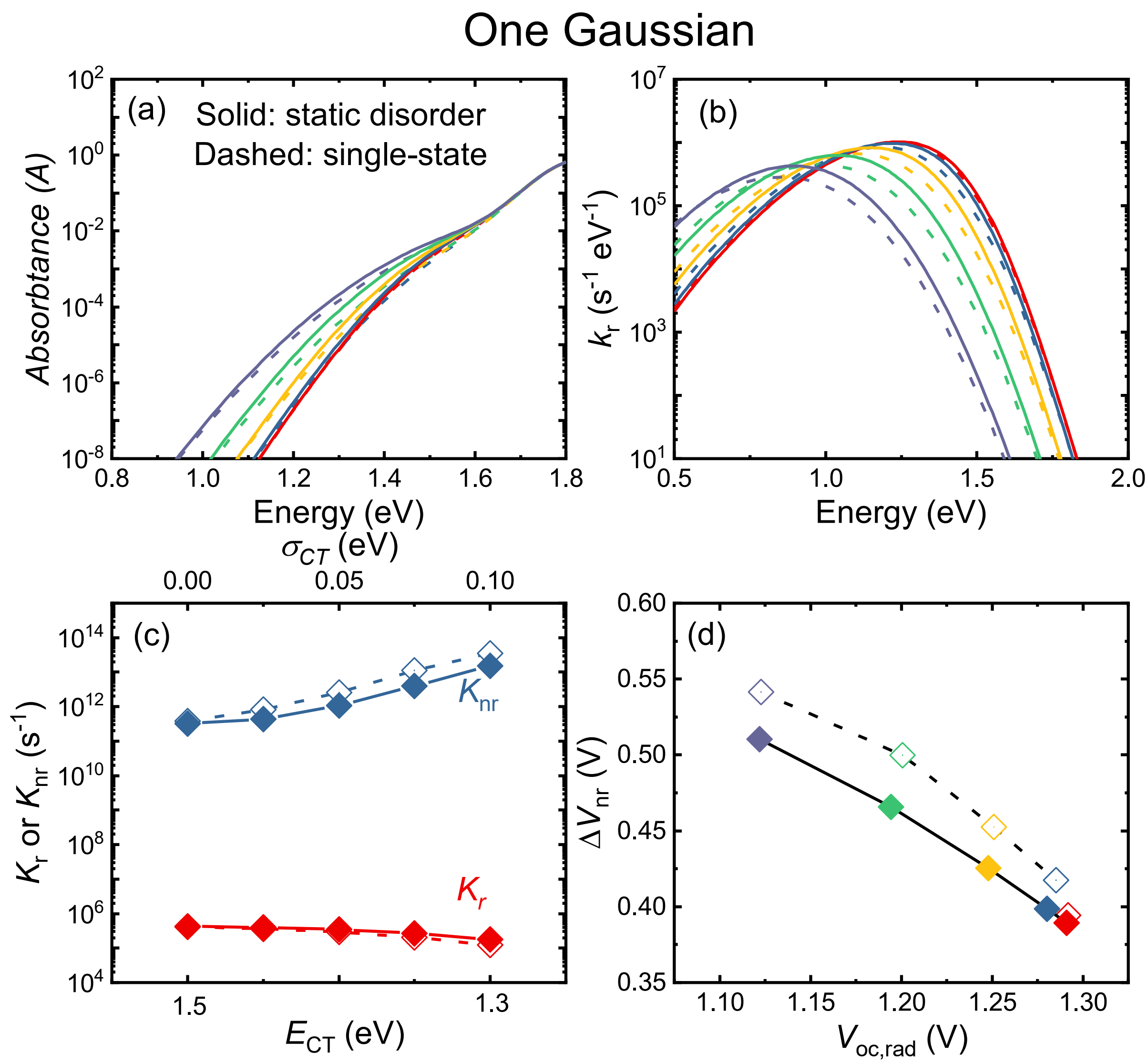

Supplementary Fig. 11. Comparison between 1 gaussian DoS model and single-state analysis. (a) absorptance, (b) emission, (c) rate constants, and (d) voltage loss plot. The input parameters for single-state analysis ($\sigma_{\mathrm{CT}} = 0$) are extracted $E_{\mathrm{CT,eff}}$ and λ_{eff} from Figure 3b.

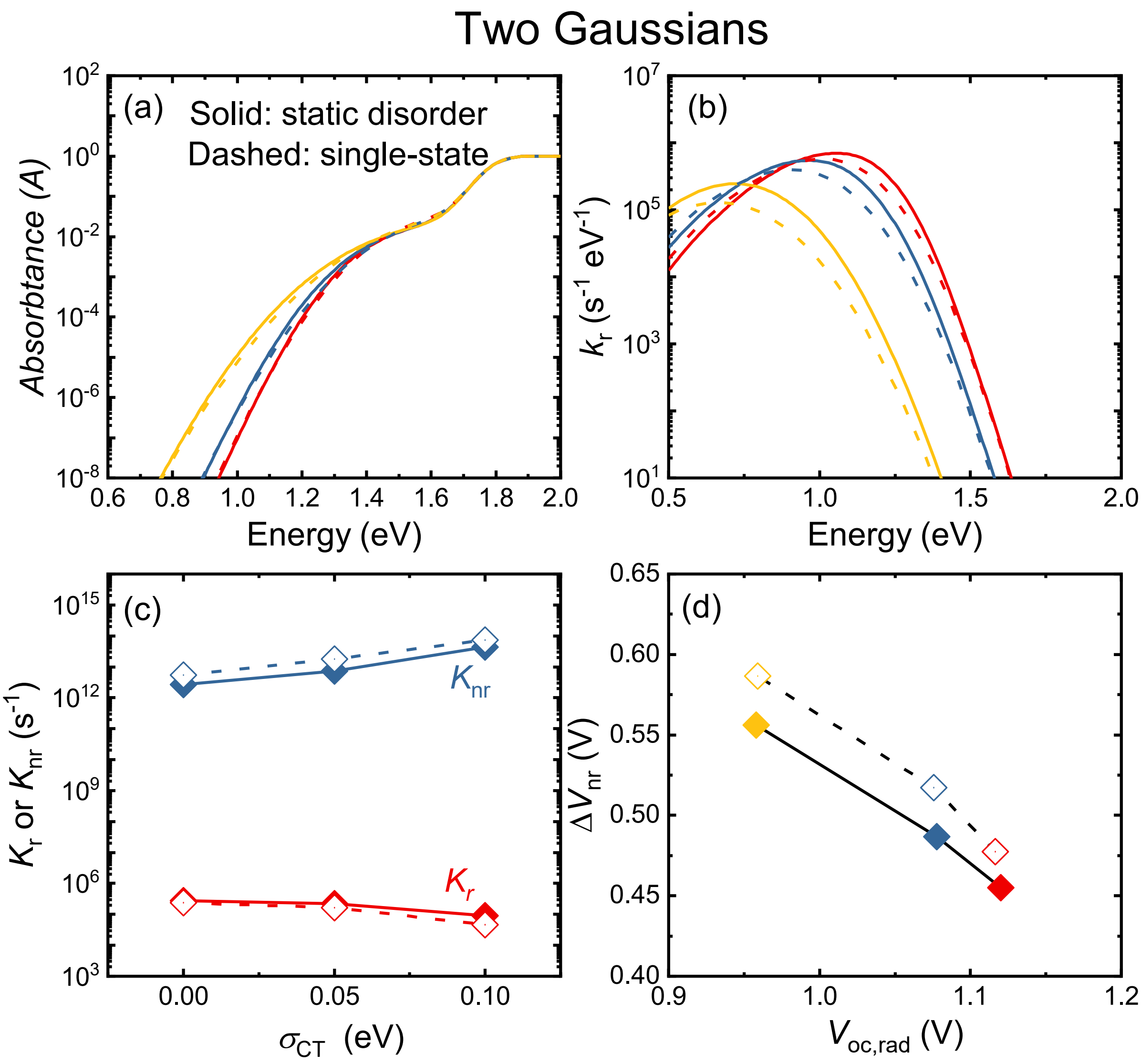

Supplementary Fig. 12. Comparison between two gaussian DoS model and single-state analysis. (a) absorptance, (b) emission, (c) rate constants, and (d) voltage loss plot. We set $E_{\mathrm{CT,C1}} = 1.5\ eV$ and $E_{\mathrm{CT,C2}} = 1.3\ eV$ with a gaussian width of σ_{CT} for both CT manifolds. σ_{CT} is varied from 0 to 0.1 eV in both CT manifolds. The rest parameters follow Supplementary Table 1.

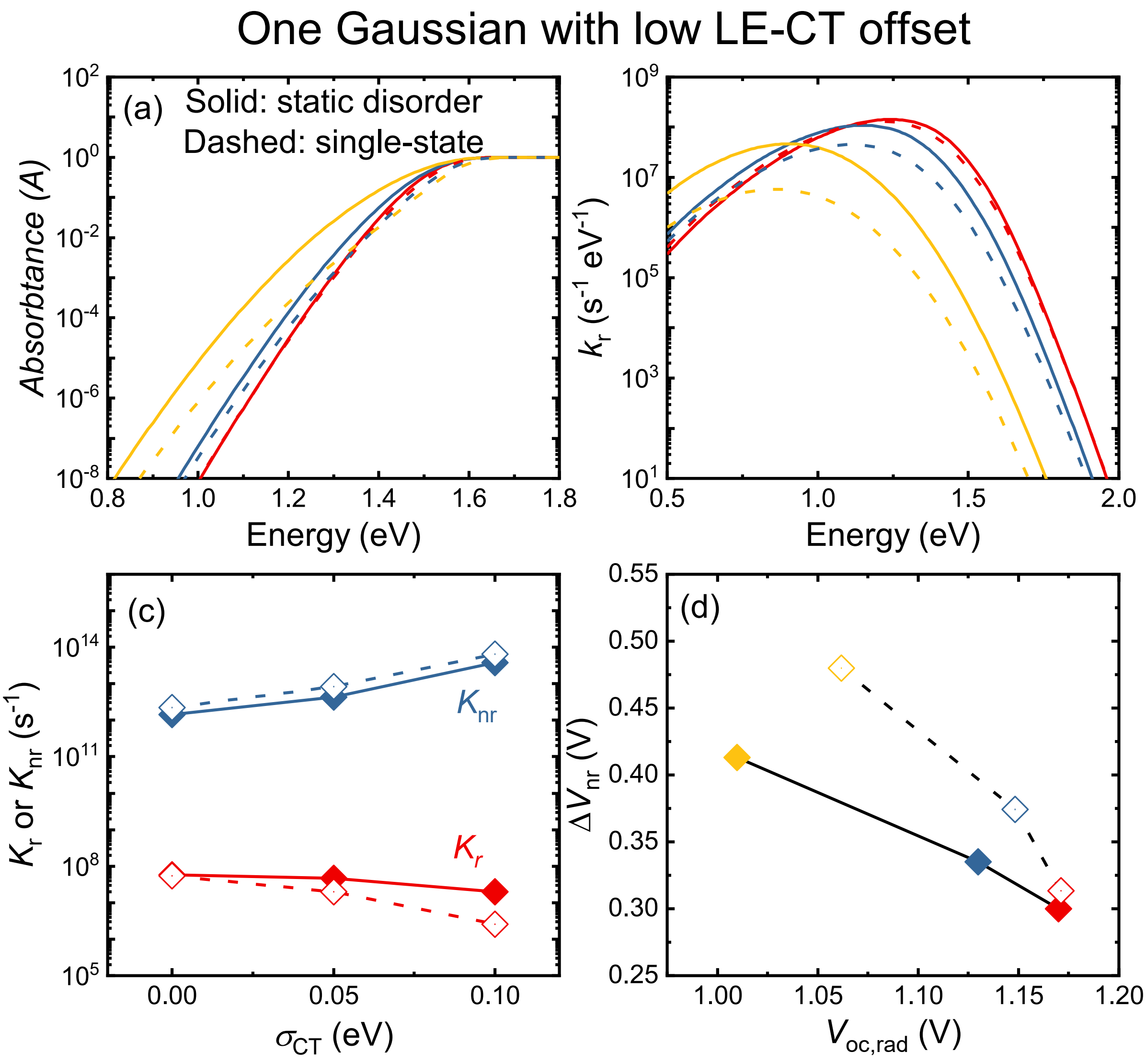

Supplementary Fig. 13. Comparison between 1 gaussian DoS model with a low LE-CT offset system (0.1 eV) and single-state analysis. (a) absorptance, (b) emission, (c) rate constants, and (d) voltage loss plot. We set $E_{\mathrm{LE}} = 1.6\ eV$ and $V_{\mathrm{LE,CT}} = 0.05\ eV$ (electronic coupling constant between LE and CT state) with the rest parameters following Supplementary Table 1. For simplicity, here we consider the effective oscillator strength for each state in the gaussian envelope is the same, and is determined by $V_{\mathrm{LE,CT}}$ and LE-CT offset[27].

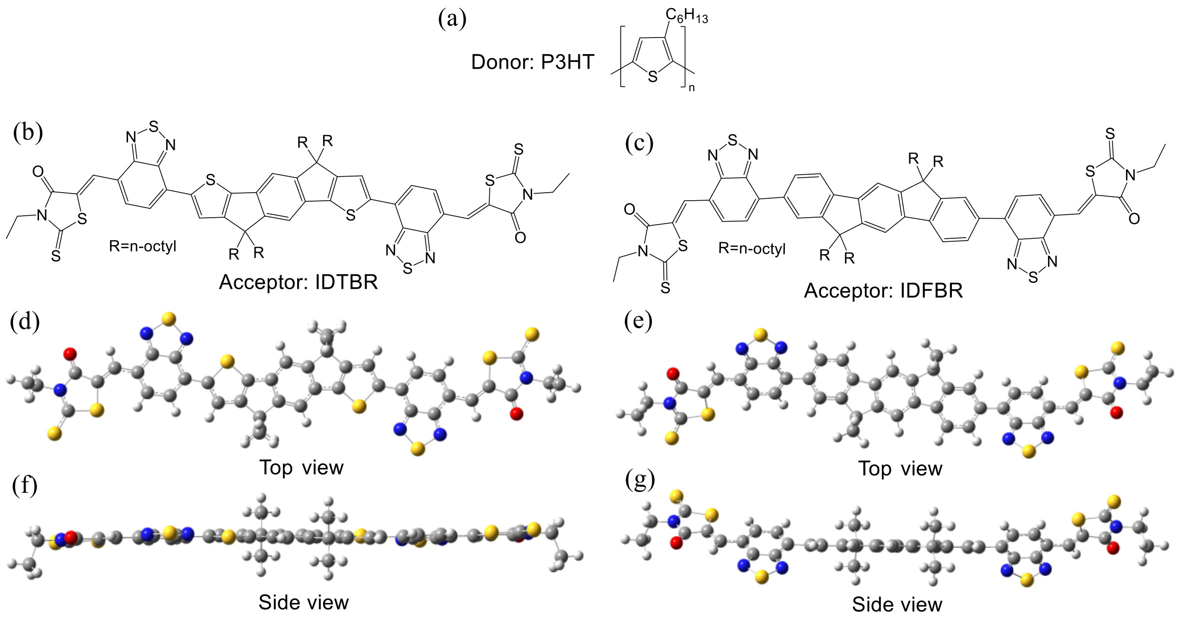

Supplementary Fig. 14. Molecular structure and optimized geometry. Molecular structure of (a) donor P3HT, (b) acceptor O-IDTBR, and (c) acceptor O-IDFBR; Optimized geometry of (d, f) O-IDTBR and (e, g) O-IDFBR in vacuum using density functional theory (DFT) with B3LYP 6-311+g(d,p). Both top (d, e) and side (f, g) views are presented. We can see that O-IDTBR and O-IDFBR have similar chemical structures but rather different backbone planarity. When blended with P3HT, the blends using planarized O-IDTBR show better crystallinity but bad mixture with P3HT, while non-planarized O-IDFBR based blends tend to be amorphous but good mixture with P3HT. Upon varying the composition, P3HT crystals remain in the O-IDTBR bends, while get disrupted easily in the O-IDFBR blends.[28]

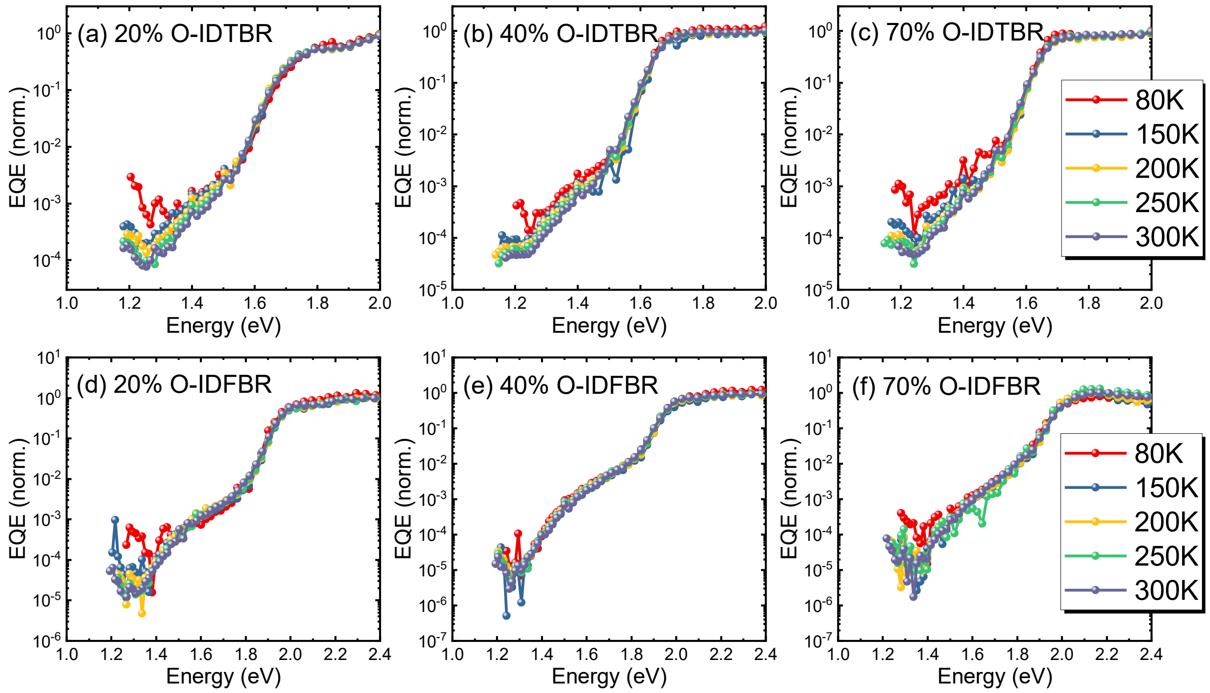

Supplementary Fig. 15. Temperature dependent EQE experiments. O-IDTBR devices with (a) 20%, (b) 40%, and (c) 70% wt%; O-IDFBR devices with (d) 20%, (e) 40%, and (f) 70% wt%. Temperature range: 80 – 300 K. Data are normalized to the absorption onset.

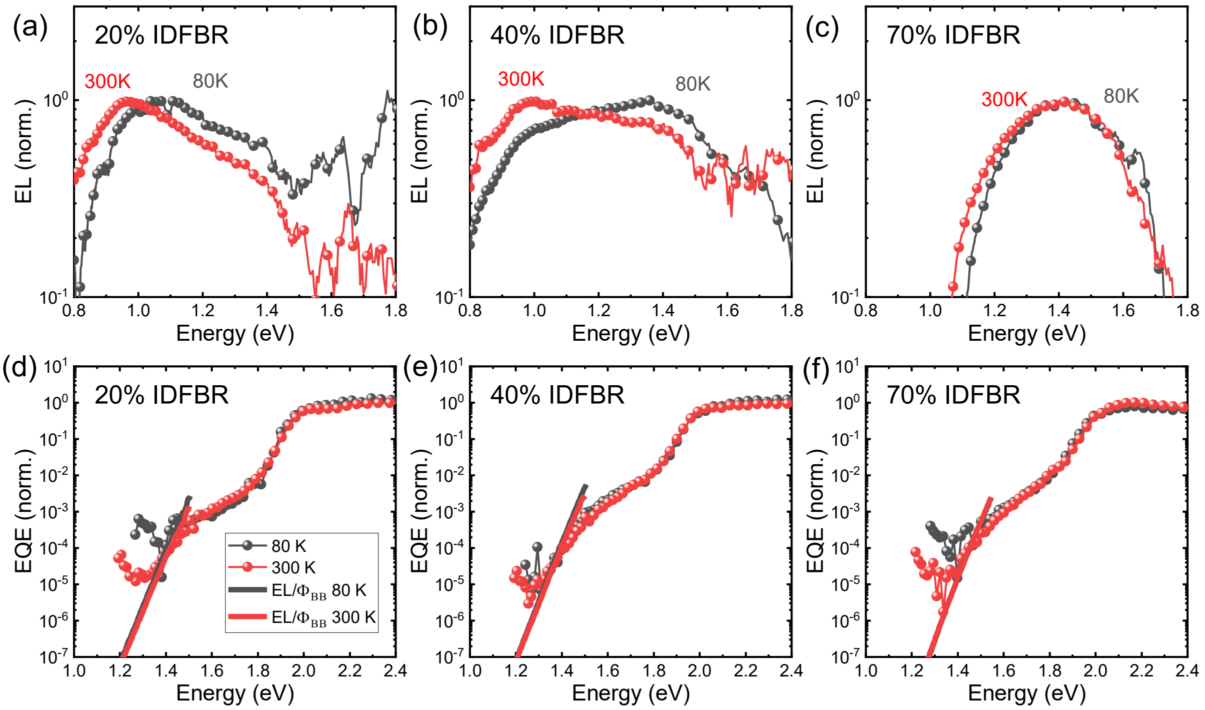

Supplementary Figure 16. Temperature dependent EL and EQE for O-IDFBR devices with different NFA wt% from 20% to 70%. (a-c) EL; (d-f) Experimental EQE and reproduced EQE using EL. Devices are measured at two different temperatures, i.e. 300K and 80K. The injection current for 20%, 40%, and 70% O-IDFBR devices are 70 mA, 50 mA and 10 mA, respectively. Reproduced EQE spectra using EL spectra based on the reciprocity relation are show in (d-f) using solid lines. It's clear that there is no clear evidence of sharpening of EL emission spectrum in consistent with the T-dependent EQE results (see also the reproduced EQE using EL, via EL/Φ_{BB}) when temperature is varied in a large range, supporting the conclusion drawn using injection-dependent EL and temperature dependent EQE that static disorder is significant in the devices we studied here. We note here that for O-IDTBR devices, we didn't manage to acquire reliable T-dependent EL data.

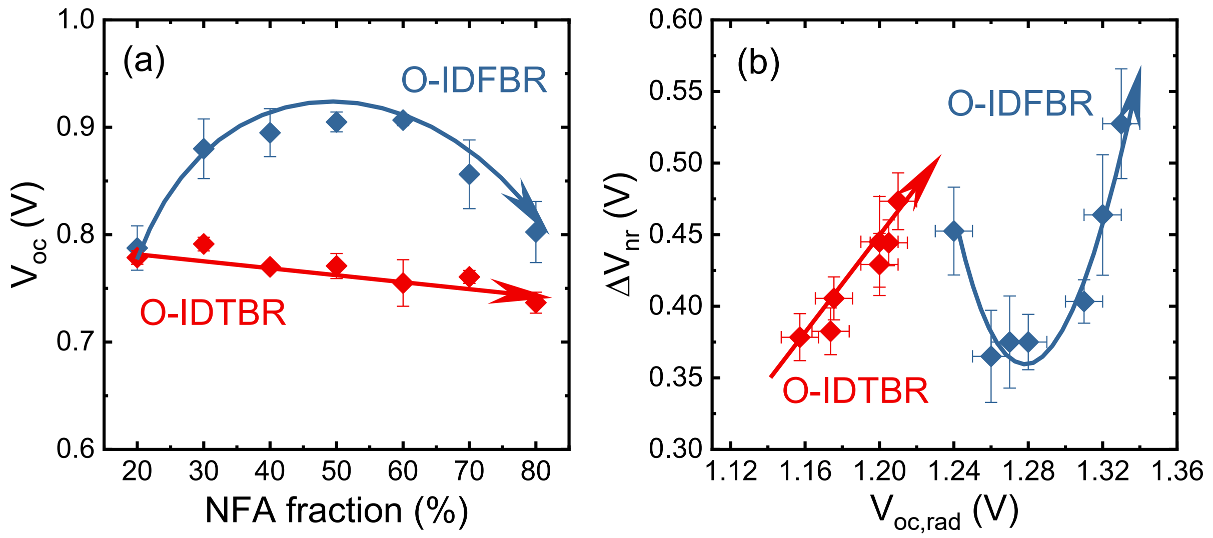

Supplementary Fig. 17. Experimental open circuit voltage and voltage losses. (a) V_{oc} as a function of NFA wt% and (b) V_{loss} plot ($\Delta V_{\mathrm{nr}}(V_{\mathrm{oc,rad}})$) at different NFA wt% for O-IDTBR and O-IDFBR based devices. Data are averaged with standard derivations indicated by error bars. Red and blue arrows are directions of increasing NFA wt% and the guides to the eye on the trends of the data points. Details for device fabrication is presented in the experimental section. The summary of the rest parameters, J_{SC}, FF, and PCE, are shown in Supplementary Fig. 18. Method for quantifying voltage loss is presented in Supplementary Method 2. Within the error bar, the V_{oc} of the O-IDTBR device decreases with increased O-IDTBR wt%, whereas for the O-IDFBR device the V_{oc} peaks at around 40-50% (wt%) O-IDFBR. The resulting ΔV_{nr} vs. $V_{\mathrm{oc,rad}}$ plots hence show contrast trends as shown in Supplementary Fig. 17b. For the O-IDTBR blends, both ΔV_{nr} and $V_{\mathrm{oc,rad}}$ increases with O-IDTBR wt%, resulting in a quasi-linear relation between ΔV_{nr} and $V_{\mathrm{oc,rad}}$. In the O-IDTBR blends, $V_{\mathrm{oc,rad}}$ increases with O-IDFBR wt% as well, however an optimum O-IDFBR wt% exists for minimum ΔV_{nr}.

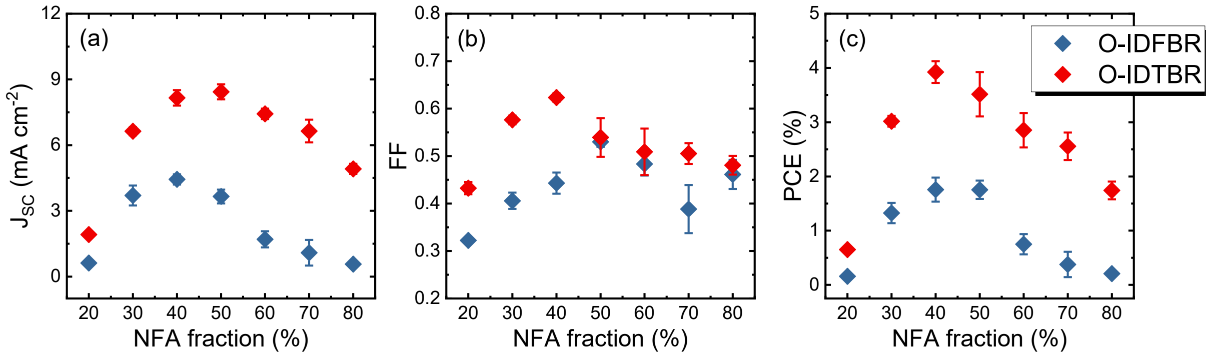

Supplementary Fig. 18. Summary of device performance. (a) Short circuit current density (J_{sc}); (b) fill factor (FF); (c) power conversion efficiency (PCE) as a function of NFA composition. Data are shown with standard derivations indicated by error bars.

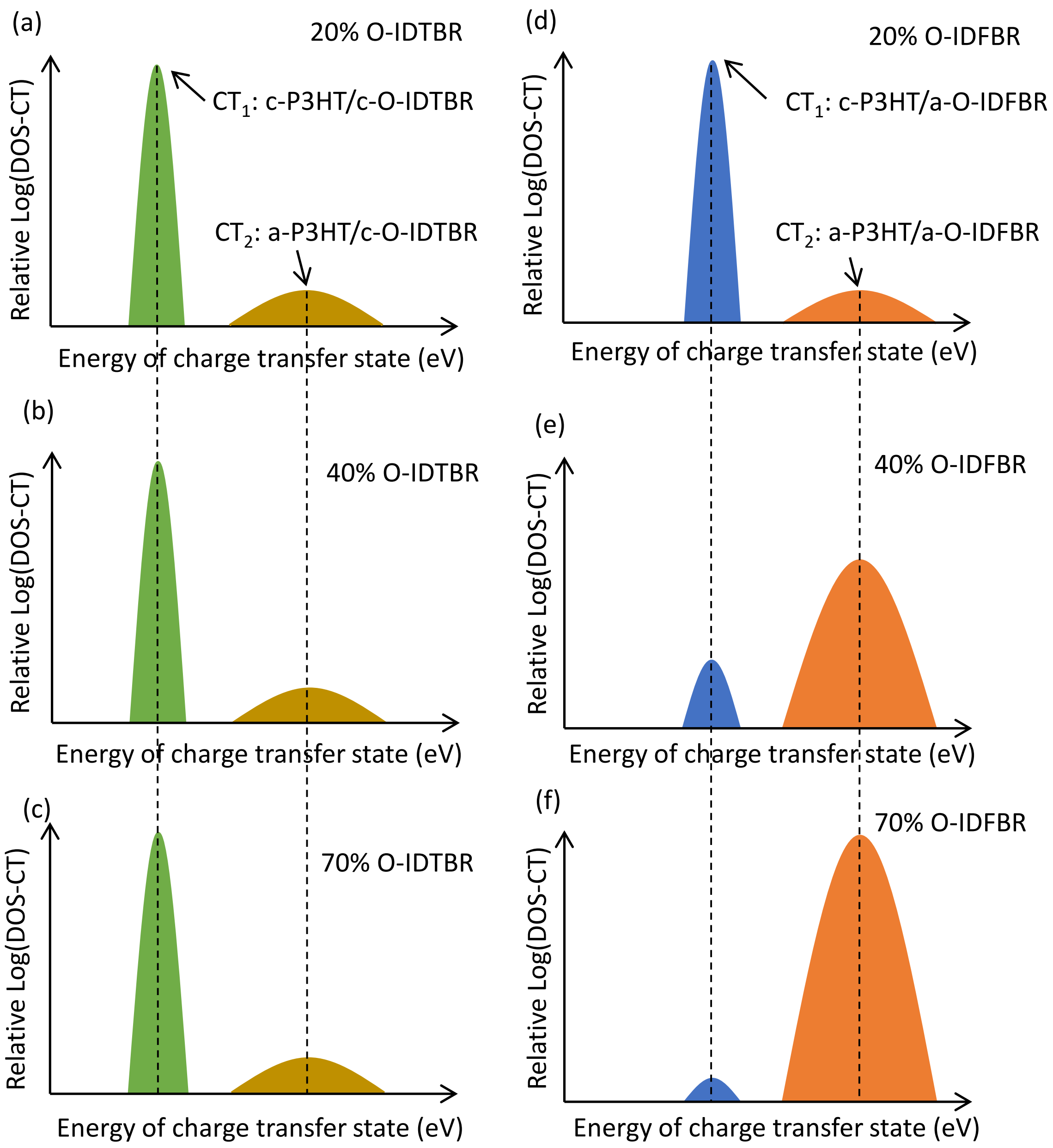

Supplementary Fig. 19. Illustration of estimated distribution of $g(E_{CT})$ as a function of composition. O-IDTBR devices with (a) 20%, (b) 40%, (c) 70% wt%, and O-IDFBR devices with (d) 20%, (e) 40%, (f) 70% wt%. Green and dark-blue shaded area represents semi-crystalline CT state, while dark-yellow and dark-orange shaded areas are amorphous CT state for O-IDTBR and O-IDFBR devices, respectively. "a-" and "c-" stand for amorphous and semi-crystalline domain, respectively.

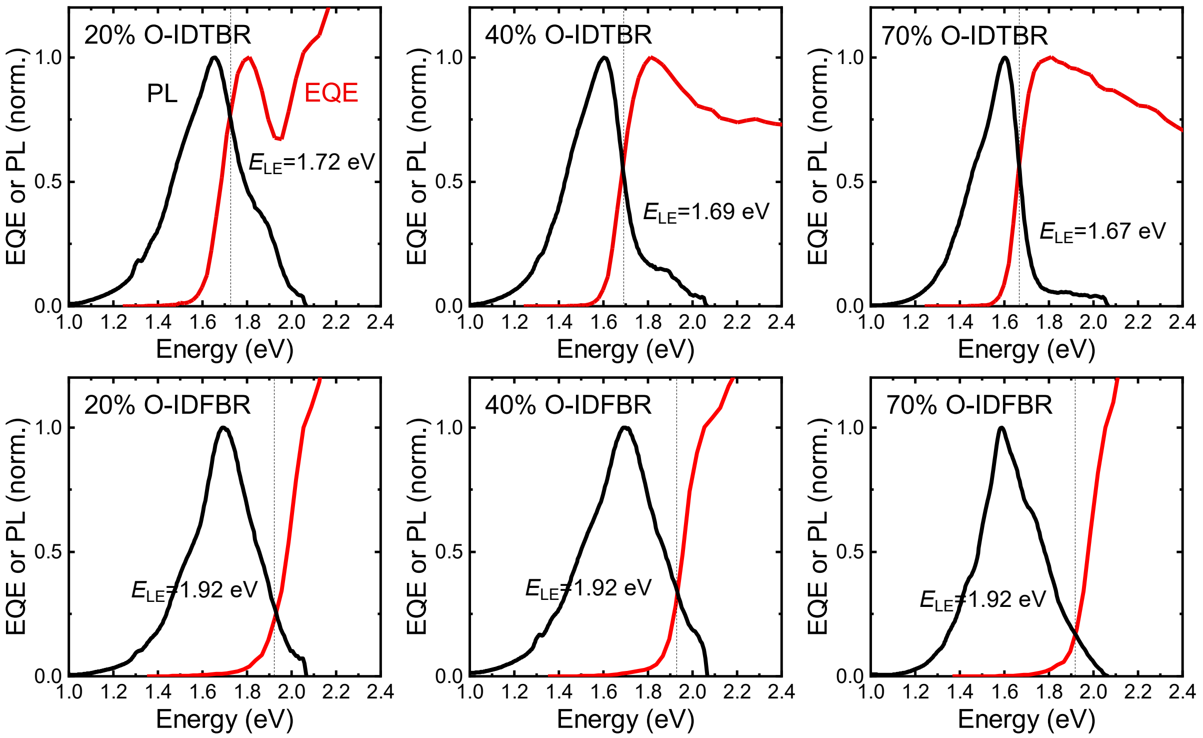

Supplementary Fig. 20. Estimated energy of local excitonic state using the intersection of normalized PL and EQE. The O-IDTBR and O-IDFBR devices with different compositions are shown.

Supplementary Note 3. Simulations of O-IDTBR devices

For the O-IDTBR devices, we propose that the position and relative density of both CT_1 and CT_2 remains constant based on the analysis in the main text. However, we suggest that f_{osc} of both CT states reduces as O-IDTBR crystal size grows upon increasing O-IDTBR wt%, based on the reduced emission intensity of CT states in Fig. 4b and previous observation[6,29]. The simulation results with changed f_{osc} are shown Supplementary Fig. 21.

Starting with injection dependent EL simulations, for all compositions, EL from CT states shows a clear two-peak transition with increased injection, which cannot be reproduced by single state model. We note here we only take account of the emission from CT states in the simulations, therefore the LE peak in experimental O-IDTBR device with 40% or 70% wt% is not reproduced.

For EQE, low injection EL, and voltage loss simulations, as shown in Fig. 6 e-g, the intensity of the EQE tail and EL emission from CT states reduce, and ΔV_{nr} increases as O-IDTBR wt% increases. This can be rationalized by the reciprocity relation, which relates emission to absorption, and the effect of high CT state emission in reducing ΔV_{nr}.

Key features of experimental observations in Fig. 4 and Fig. 5 for the O-IDTBR devices have been reproduced considering the change of f_{osc} only.

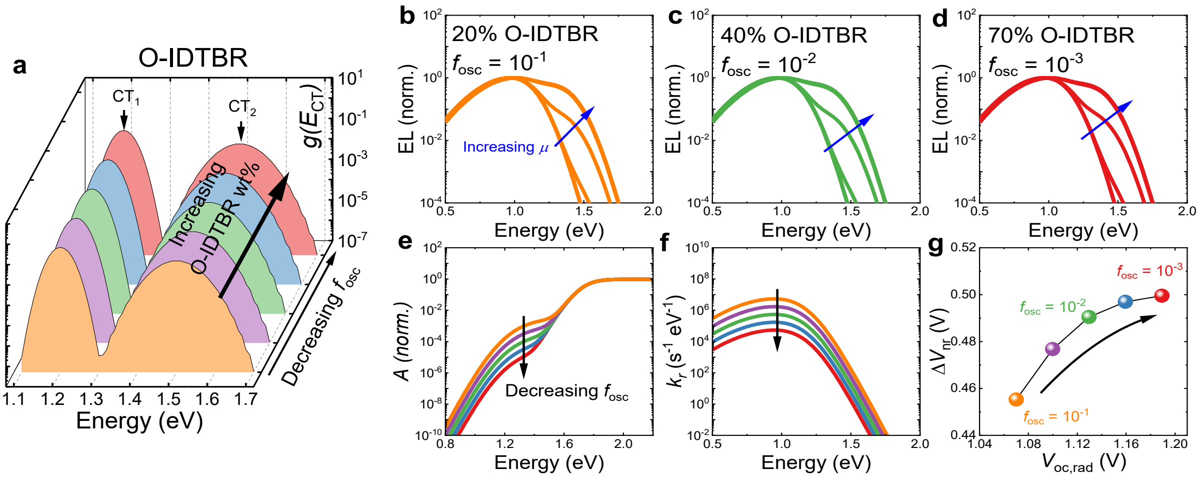

Supplementary Fig. 21. Estimated distribution of density of CT state $g(E_{\mathrm{CT}})$, and simulated EQE, EL and V_{loss} for the O-IDTBR devices. Illustration of the distribution of $g(E_{\mathrm{CT}})$ in (**a**) the O-IDTBR devices used in the model. In (**a**), the trend in $g(E_{\mathrm{CT}})$ with increasing O-IDTBR fraction from 20 to 70wt% is modelled via a decrease on the oscillator strength f_{osc} of both CT states from 10^{-1} to 10^{-3}. $g(E_{\mathrm{CT}})$ is modelled using of two CT manifolds with a gaussian width of 0.02 eV (semi-crystalline CT_1) and 0.04 eV (amorphous CT_2), respectively. The total $g(E_{\mathrm{CT}})$ is a superposition of CT_1 and CT_2. Parts **b-d** show simulated and normalized injection dependent emission spectra with different NFA wt%. Blue arrows are indications of increasing μ in the range of 1-2 eV. Parts **e-g** show absorptance (A), rate constant of emission per photon energy (k_r), and $\Delta V_{\mathrm{nr}}(V_{\mathrm{oc,rad}})$ plot. We note here that the emission from LE state hasn't been simulated as for large D-A HOMO-HOMO offset system (i.e. ~0.5 eV for P3HT:O-IDTBR) the contribution from LE state to total recombination is negligible.[30] The reason that we can see clear LE state emission from O-IDTBR excitons is because of the large amount of O-IDTBR cluster in the case of high O-IDTBR weight fraction in the blend (i.e. 40% and 70%), this leads to the emission from "isolated" O-IDTBR clusters, which however doesn't contribute to the CT states at all, as they decay directly without charge transfer processes. The detailed parameters used to model the O-IDTBR devices are listed in Supplementary Table 6.

Supplementary Note 4. Injection dependent EL simulations

In the injection dependent electroluminescence simulations, the emission spectra are changing with increased injection (in both experiments in Fig. 5 and simulations in Fig. 6), which is a signature of a departure from quasi-thermal equilibrium (QTE) condition under high injection condition. That means the emission spectra are no longer describable by the QTE theory we introduced in the theory section (Eq. (11) in the main text), but in fact reflect a kinetically limited state filling effect[31]. In previous work, Gong et al.[31] addressed the problem by considering the bias dependent distributions of the electrons and holes that contribute to the EL, using Fermi-Dirac (FD) statistics. In a different approach, Burke et al.[22] argued that the limited capacity of interface states for excitons meant that the CT states should obey FD statistics. Here, we adopt an approach similar to Burke and use the formalism below to model the EL data:

$$\mathrm{EL}(\hbar\omega) = \frac{1}{Z_{\mathrm{rec}}}\sum_{t}\int_{a}^{b}\frac{1}{3\pi\epsilon_0\hbar^4}\left(\frac{\hbar\omega}{c}\right)^3 M_t(E_{\mathrm{CT}})^2\,\mathrm{FCWD}_{\mathrm{rec},t}(\hbar\omega, E_{\mathrm{CT}})\,c_t D_t(E_{\mathrm{CT}})\frac{1}{\exp\left(\frac{E_{\mathrm{CT}}-\mu}{k_B T}\right)+1}\,d(E_{\mathrm{CT}})\,. \quad (24)$$

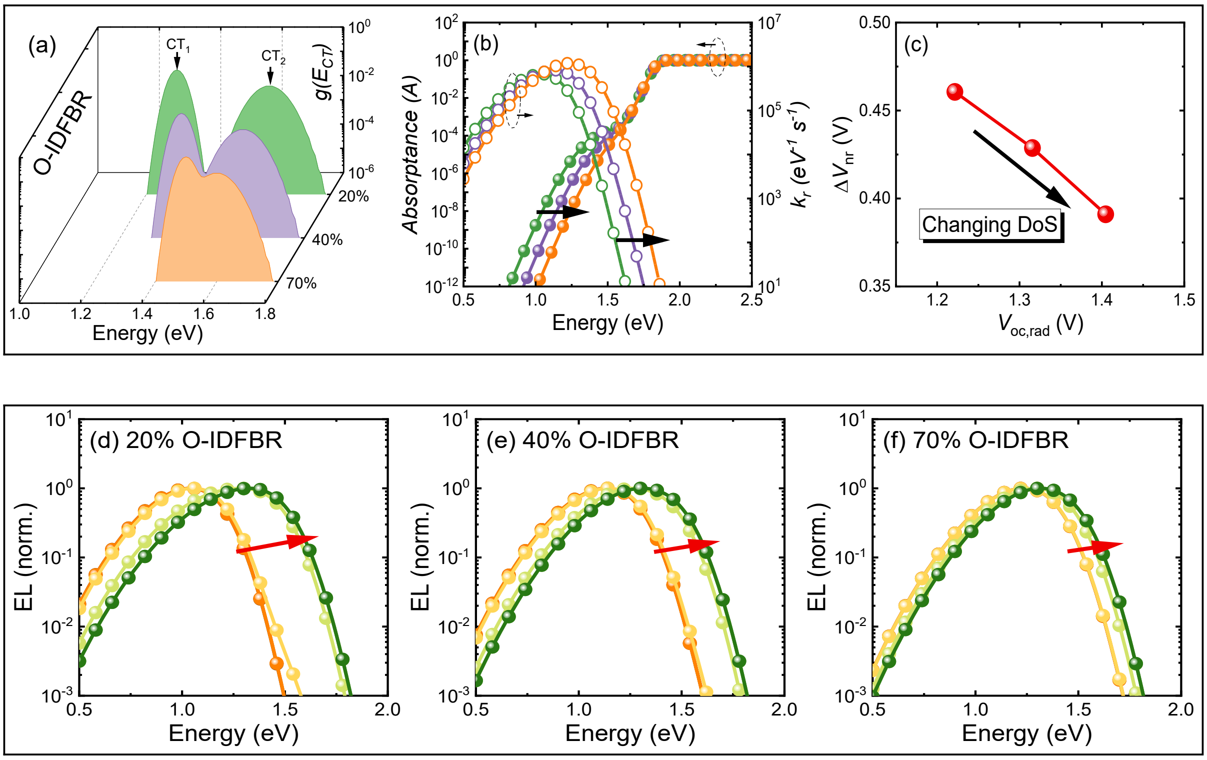

Supplementary Fig. 22. Effects of the change of energy position of CT₁ on emission at low and different injection conditions, absorption spectrum, and voltage loss. $E_{\mathrm{CT,2}}$ is set to 1.6 eV, while $E_{\mathrm{CT,1}}$ is changed from 1.3 to 1.5 eV. We see that in Supplementary Fig. 22b the emission spectrum shifts to the blue and the absorption tail becomes sharper upon moving CT_1 to higher energy. And the voltage loss plot shows a similar trend as predicted in Figure 6 e-g as well as the experimental results (Figure 4d-f) in the main text. We also see similar simulated injection dependent emission spectrums as a function of composition in the bottom panel (Supplementary Fig. 22 d-f) as shown in Figure 6 b-d.

Supplementary Note 5. Commenting on the non-quasi-thermal equilibrium site distribution of CT states:

The validity of quasi-thermal equilibrium (QTE) condition in working organic photovoltaics under illumination or under a bias has been an assumption that has underpinned the results of most voltage loss and emission studies[3,32], but has come under scrutiny in recent papers[20,33]. The argument is based on the disordered nature of organic semiconductor induced spatially separated CT states, such that the chance for recombination happening at the lowest CT states is low[33], and implicitly they consider that those spatially separated CT states are not coupled, and different chemical potential energies for different states are expected under non-QTE condition. This leads to a modified expression for the recombination rate constants and Z factor for recombination, as follows:

$$K_{\mathrm{nr}} = \frac{1}{Z_{\mathrm{rec}}}\sum_{t}\int_{a}^{b}\frac{2\pi}{\hbar}V_t(E_{\mathrm{CT}})^2\mathrm{FCWD}_{\mathrm{rec},t}(0,E_{\mathrm{CT}})c_tD_t(E_{\mathrm{CT}})\exp\left(-\frac{E_{\mathrm{CT}}-\mu_t}{k_BT}\right)d(E_{\mathrm{CT}}), (25)$$

$$k_r(\hbar\omega) = \frac{1}{Z_{\mathrm{rec}}}\sum_{t}\int_{a}^{b}\frac{1}{3\pi\epsilon_0\hbar^4}\left(\frac{\hbar\omega}{c}\right)^3 M_t(E_{\mathrm{CT}})^2\mathrm{FCWD}_{\mathrm{rec},t}(\hbar\omega,E_{\mathrm{CT}})c_tD_t(E_{\mathrm{CT}})\exp\left(-\frac{E_{\mathrm{CT}}-\mu_t}{k_BT}\right)d(E_{\mathrm{CT}}), (26)$$

$$Z_{\mathrm{rec}} = \sum_{t}\left\{\sum_{m=0}^{\infty}\exp\left(-\frac{m\hbar\Omega_t}{k_BT}\right)\int_{a}^{b}c_tD_t(E_{\mathrm{CT}})\exp\left(-\frac{E_{\mathrm{CT}}-\mu_t}{k_BT}\right)d(E_{\mathrm{CT}})\right\}. (27)$$

Here we discuss the effect of non-QTE condition on the accuracy of our analysis of experimental results. To model the scenario of non-QTE, we consider that each of the observed two CT manifolds in the experimental section has an individual chemical potential energy μ_1 and μ_2 ($\mu_1 \neq \mu_2$). As an example of presenting the effect of non-QTE site distribution, we set $\mu_1 = 0$, and vary μ_2 in the range of $[-0.5, 0.5]\ eV$, and the results are shown in Supplementary Fig. 23. We first notice that the principle of detailed balance is no longer valid if $\mu_1 \neq \mu_2$. Upon increasing μ_2 we see the contribution from CT_2 manifold becomes higher in the emission spectrum and dominates the spectrum with $\mu_2 > 0.2\ eV$, as shown in Supplementary Fig. 23a and b. However, the change on the emission spectrum is able to affect the rate constant of radiative and non-radiative decay only when the chemical potential energy of CT_2 manifold is significantly larger than CT_1, i.e. $\mu_2 > 0.2\ eV\ (7.7k_BT)$ (Supplementary Fig. 23c), hence the voltage losses (Supplementary Fig. 23d). Since we see that under low injection condition EL emission preferably comes from CT_1 manifold from the experimental results in this work (See Figure 5), the possibility that the devices are largely off from QTE condition is low, and only a small chemical potential energy difference between two CT manifolds is possible, which however has negligible impact on the emission spectrum, hence the voltage loss results. Therefore, we believe assuming QTE condition is a safe approach in this study. Yet, more studies are needed for a comprehensive understanding on the impact of non-QTE site distribution in OPV.

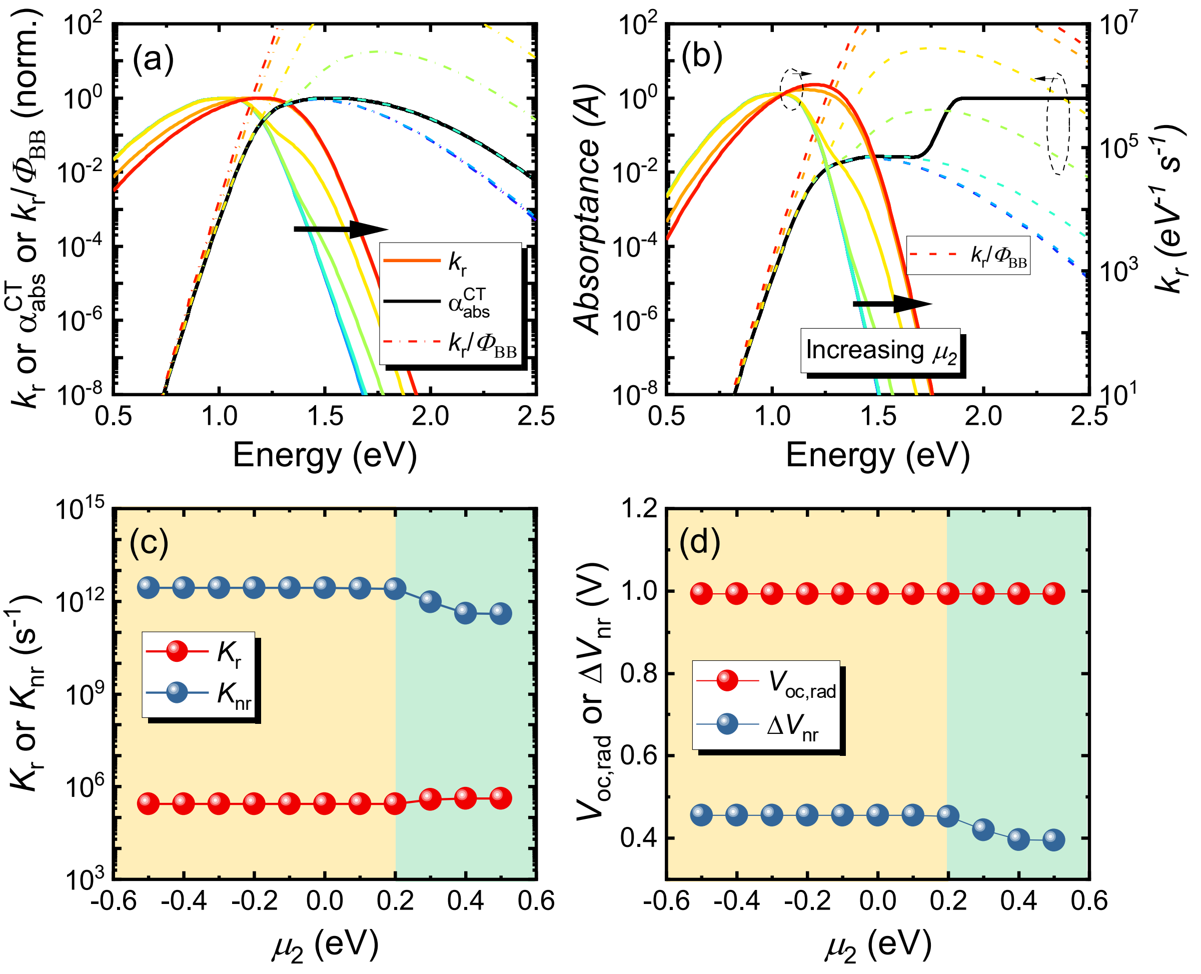

Supplementary Fig. 23. Effect of non-QTE site distribution on emission, absorption, rate constant of decay, and voltage loss. (a) Normalized rate constant per photon energy of emission (k_r), absorption coefficient of CT states ($\alpha_{\mathrm{abs}}^{\mathrm{CT}}$), and k_r/ϕ_{BB}; (b) Normalized rate constant per photon energy of emission (k_r), absorptance (A) and k_r/ϕ_{BB}; (c) rate constant of radiative (K_r) and nonradiative (K_{nr}) transition as a function of μ_2; (d) ΔV_{nr} and $V_{\mathrm{oc,rad}}$ as a function of μ_2.

Supplementary Table 1. Key parameter values used in Figure 2a-d

Parameter	Value	Units
Difference in the static dipole moment ($\left\|\overrightarrow{\Delta\mu}\right\|$)	5	Debye
Oscillator strength (f_{osc})	1×10^{-2}	Unitless
High frequency reorganisation energy (λ_i)	0.2	eV
Low frequency reorganisation energy (λ_o)	0.2	eV
Vibrational mode harmonic oscillator energy ($\hbar\Omega$)	0.15	eV
refractive index (n)	1.5	Unitless
The ratio of CT state density ($N_{\mathrm{CT/LE}}$)	1	Unitless
Energy of local excitonic state (E_{LE})	1.8	eV
Energy of the centre of CT states ($E_{\mathrm{CT},c}$)	1.5	eV
$g(E_{\mathrm{CT}})$ Gaussian width (σ_{CT})	0.0-0.1	eV

Note: light yellow shaded area indicates the variable(s).

Supplementary Table 2. Key parameter values used in Figure 2e-h

Parameter	Value	Units
Difference in the static dipole moment ($\left\|\overrightarrow{\boldsymbol{\Delta\mu}}\right\|$)	5	Debye
Oscillator strength (f_{osc})	1×10^{-2}	Unitless
High frequency reorganisation energy (λ_i)	0.2	eV
Low frequency reorganisation energy (λ_o)	0.2	eV
Vibrational mode harmonic oscillator energy ($\hbar\Omega$)	0.15	eV
refractive index (n)	1.5	Unitless
The ratio of CT state density ($N_{\mathrm{CT/LE}}$)	1	Unitless
Energy of local excitonic state (E_{LE})	1.8	eV
Energy of the centre of CT states ($E_{\mathrm{CT,C1}}$)	1.2	eV
Energy of the centre of CT states ($E_{\mathrm{CT,C2}}$)	1.5	eV
Gaussian width for both CT manifold ($\sigma_{\mathrm{CT,C1}}$ and $\sigma_{\mathrm{CT,C2}}$)	0.0-0.1	eV

Note: light yellow shaded area indicates the variable(s). For simplicity, we assume CT_1 and CT_2 manifold share the same set of parameters, except for the peak energies.

Supplementary Table 3. Key parameter values used in Figure 2i-l

Parameter	Value	Units
Difference in the static dipole moment ($\lvert\overrightarrow{\Delta\mu}\rvert$)	5	Debye
Oscillator strength (f_{osc})	1×10^{-2}	Unitless
High frequency reorganisation energy (λ_i)	0.2	eV
Low frequency reorganisation energy (λ_o)	0.2	eV
Vibrational mode harmonic oscillator energy ($\hbar\Omega$)	0.15	eV
refractive index (n)	1.5	Unitless
The ratio of CT state density ($N_{\mathrm{CT/LE}}$)	1	Unitless
Energy of local excitonic state (E_{LE})	1.8	eV
Energy of the centre of CT states ($E_{\mathrm{CT,C1}}$)	1-1.4	eV
Energy of the centre of CT states ($E_{\mathrm{CT,C2}}$)	1.5	eV
Gaussian width for both CT manifold ($\sigma_{\mathrm{CT,C1}}$ and $\sigma_{\mathrm{CT,C2}}$)	0.05	eV

Note: light yellow shaded area indicates the variable(s). For simplicity, we assume CT_1 and CT_2 manifold share the same set of parameters, except for the peak energies.

Supplementary Table 4. Key parameter values used in **Supplementary Fig. 4-8**

Parameter	Value when it's constant	Value when it's varied	Units
Difference in the static dipole moment ($\lvert\overrightarrow{\Delta\mathbf{\mu}}\rvert$)	5	N/A	Debye
Oscillator strength (f_{osc})	1×10^{-2}	$[10^{-2}, 1]$	Unitless
High frequency reorganisation energy (λ_i)	0.2	$[0.05, 0.2]$	eV
Low frequency reorganisation energy (λ_o)	0.2	$[0.05, 0.2]$	eV
Vibrational mode harmonic oscillator energy ($\hbar\Omega$)	0.15	N/A	eV
refractive index (n)	1.5	N/A	Unitless
The ratio of CT state density ($N_{\mathrm{CT/LE}}$)	1	$[10^{-3}, 1]$	Unitless
Energy of local excitonic state (E_{LE})	1.8	N/A	eV
Energy of the centre of CT states ($E_{\mathrm{CT},C}$)	1.5	$[1.5, 1.65]$	eV
$g(E_{\mathrm{CT}})$ Gaussian width (σ_{CT})	0 or 0.1	N/A	eV

Note: the values in square brackets give the range of changing parameter values. To produce Supplementary Fig. 4-8, when we perform simulations on one varied parameter, for example, $E_{\mathrm{CT},C}$*, we assume other parameter are constant, which are listed in the second column ("Value when it's constant"). Light yellow shaded area indicates the variable(s).*

Supplementary Table 5. Key parameter values to calculate voltage and efficiency limit with 1 gaussian

Parameter	Values	Units
Difference in the static dipole moment ($\lvert\overrightarrow{\Delta\boldsymbol{\mu}}\rvert$)	30	Debye
Oscillator strength ($f_{\rm osc}$)	1	Unitless
High frequency reorganisation energy (λ_i)	0.1	eV
Low frequency reorganisation energy (λ_o)	0.1	eV
Vibrational mode harmonic oscillator energy ($\hbar\Omega$)	0.15	eV
refractive index (n)	1.5	Unitless
The ratio of CT state density ($N_{\rm CT/EX}$)	1	Unitless
Energy of local excitonic state ($E_{\rm LE}$)	1.4	eV
Energy of the centre of CT states ($E_{{\rm CT},C}$)	1.4	eV
$g(E_{\rm CT})$ Gaussian width ($\sigma_{\rm CT}$)	0.0-0.1	eV

Note: light yellow shaded area indicates the variable(s).

Supplementary Table 6. Key parameter values to model experimental devices in Fig. 6

Parameter	O-IDTBR		O-IDFBR		Units
	CT_1	CT_2	CT_1	CT_2	
Difference in the static dipole moment ($\lvert\overrightarrow{\Delta\mu}\rvert$)	5				Debye
High frequency reorganisation energy (λ_i)	0.2				eV
Low frequency reorganisation energy (λ_o)	0.2				eV
Vibrational mode harmonic oscillator energy ($\hbar\Omega$)	0.15				eV
refractive index (n)	1.5				Unitless
The ratio of CT state density ($N_{\mathrm{CT/LE}}$)	0.01				Unitless
Energy of local excitonic state (E_{LE})	1.7		1.9		eV
Oscillator strength (f_{osc})	[10^{-3}, 10^{-1}]		10^{-3}	10^{-1}	Unitless
The density fraction of CT state (F_C)	0.7	0.3	[0.01, 0.7]	0.3	Unitless
Energy of the centre of CT states ($E_{\mathrm{CT},C}$)	1.2	1.5	1.3	1.5	eV
CT DOS width (σ_{CT})	0.02	0.04	0.02	0.04	eV

Note: E_{LE} is estimated using Supplementary Fig. 20, and for simplicity in the modelling we assume it's unchanged while changing the composition as it's not the determining factor. The range of f_{osc} was chosen based on the change of the relative CT state emission intensity from Figure 4b, where roughly two orders of magnitude change was seen. The range of F_{C1} was chosen based on the estimated interfacial density fraction changed based on Ref. [28], where ~70% of crystalline interface was observed in the case of 20% O-IDFBR devices. This value was significantly reduced when more O-IDFBR was added in the blend. We here chose to have roughly two orders of magnitude changes to see the effect clearly.

Supplementary References

1. Blank, B., Kirchartz, T., Lany, S. & Rau, U. Selection metric for photovoltaic materials screening based on detailed-balance analysis. *Phys. Rev. Appl.* **8**, 024032 (2017).

2. Rau, U. Reciprocity relation between photovoltaic quantum efficiency and electroluminescent emission of solar cells. *Phys. Rev. B* **76**, 085303 (2007).

3. Yao, J. *et al.* Quantifying Losses in Open-Circuit Voltage in Solution-Processable Solar Cells. *Phys. Rev. Appl.* **4**, 014020 (2015).

4. Rau, U., Blank, B., Müller, T. C. M. & Kirchartz, T. Efficiency Potential of Photovoltaic Materials and Devices Unveiled by Detailed-Balance Analysis. *Phys. Rev. Appl.* **7**, 044016 (2017).

5. Shockley, W. & Queisser, H. J. Detailed Balance Limit of Efficiency of p-n Junction Solar Cells. *J. Appl. Phys.* **32**, 510–519 (1961).

6. Azzouzi, M. *et al.* Nonradiative Energy Losses in Bulk-Heterojunction Organic Photovoltaics. *Phys. Rev. X* **8**, 031055 (2018).

7. Vandewal, K., Tvingstedt, K., Gadisa, A., Inganäs, O. & Manca, J. V. Relating the open-circuit voltage to interface molecular properties of donor:acceptor bulk heterojunction solar cells. *Phys. Rev. B - Condens. Matter Mater. Phys.* **81**, (2010).

8. Ridley, B. K. *Quantum processes in semiconductors*. (Oxford University Press, 2013).

9. George C. Schatz, M. A. R. *Quantum mechanics in chemistry*. (Courier Corporation, 1993).

10. Bhattacharya, D., Vaval, N. & Pal, S. Electronic transition dipole moments and dipole oscillator strengths within Fock-space multi-reference coupled cluster framework: An efficient and novel approach. *J. Chem. Phys.* **138**, 094108 (2013).

11. Fox, M. *Optical Properties of Solids*. *Oxford University Press* (2001).

12. Huang, K. & Rhys, A. Theory of Light Absorption and Non-Radiative Transitions in F-Centres. *Proc. R. Soc. A Math. Phys. Eng. Sci.* **204**, 406–423 (1950).

13. Benduhn, J. *et al.* Intrinsic non-radiative voltage losses in fullerene-based organic solar cells. *Nat. Energy* **2**, 1–23 (2017).

14. Griffiths, D. J. & Schroeter, D. F. *Introduction to Quantum Mechanics*. *Introduction to Quantum Mechanics* (Cambridge University Press, 2018). doi:10.1017/9781316995433

15. Nitzan, A., Mukamel, S. & Jortner, J. Energy gap law for vibrational relaxation of a molecule in a dense medium. *J. Chem. Phys.* **63**, 200–207 (1975).

16. Zheng, J., Kang, Y. K., Therien, M. J. & Beratan, D. N. Generalized Mulliken-Hush analysis of electronic coupling interactions in compressed ??-stacked porphyrin-bridge-quinone systems. *J. Am. Chem. Soc.* **127**, 11303–11310 (2005).

17. Azzouzi, M. *Voltage Losses and Recombination Mechanisms in Organic Solar Cells*. *Imperial College London* (2020).

18. Kahle, F.-J. J., Rudnick, A., Bässler, H. & Köhler, A. How to interpret absorption and fluorescence spectra of charge transfer states in an organic solar cell. *Mater. Horizons* **5**, 837–848 (2018).

19. Linderl, T. *et al.* Crystalline versus Amorphous Donor-Acceptor Blends: Influence of Layer Morphology on the Charge-Transfer Density of States. *Phys. Rev. Appl.* **13**, 024061 (2020).

20. Felekidis, N., Melianas, A. & Kemerink, M. The Role of Delocalization and Excess Energy in the Quantum Efficiency of Organic Solar Cells and the Validity of Optical Reciprocity Relations. *J. Phys. Chem. Lett.* **11**, 3563–3570 (2020).

21. Zheng, Z., Tummala, N. R., Wang, T., Coropceanu, V. & Brédas, J. L. Charge-Transfer States at Organic–Organic Interfaces: Impact of Static and Dynamic Disorders. *Adv. Energy Mater.* **9**, 1–7 (2019).

22. Burke, T. M., Sweetnam, S., Vandewal, K. & McGehee, M. D. Beyond Langevin recombination: How equilibrium between free carriers and charge transfer states determines the open-circuit voltage of organic solar Cells. *Adv. Energy Mater.* **5**, 1500123 (2015).

23. Ullbrich, S. *et al.* Emissive and charge-generating donor–acceptor interfaces for organic optoelectronics with low voltage losses. *Nat. Mater.* **18**, 459–464 (2019).

24. Li, S. *et al.* Highly Efficient Fullerene-Free Organic Solar Cells Operate at Near Zero Highest Occupied Molecular Orbital Offsets. *J. Am. Chem. Soc.* **141**, 3073–3082 (2019).

25. Wang, R. *et al.* Rational Tuning of Molecular Interaction and Energy Level Alignment Enables High-Performance Organic Photovoltaics. *Adv. Mater.* **31**, 1904215 (2019).

26. Yuan, J. *et al.* Single-Junction Organic Solar Cell with over 15% Efficiency Using Fused-Ring Acceptor with Electron-Deficient Core. *Joule* **3**, 1140–1151 (2019).

27. Eisner, F. D. F. D. *et al.* Hybridization of Local Exciton and Charge-Transfer States Reduces Nonradiative Voltage Losses in Organic Solar Cells. *J. Am. Chem. Soc.* **141**, 6362–6374 (2019).

28. Rezasoltani, E. *et al.* Correlating the Phase Behavior with the Device Performance in Binary Poly-3-hexylthiophene: Nonfullerene Acceptor Blend Using Optical Probes of the Microstructure. *Chem. Mater.* **32**, 8294–8305 (2020).

29. Liu, X., Ding, K., Panda, A. & Forrest, S. R. Charge Transfer States in Dilute Donor-Acceptor Blend Organic Heterojunctions. *ACS Nano* **10**, 7619–7626 (2016).

30. Classen, A. *et al.* The role of exciton lifetime for charge generation in organic solar cells at negligible energy-level offsets. *Nat. Energy* **5**, 711–719 (2020).

31. Gong, W. *et al.* Influence of energetic disorder on electroluminescence emission in polymer: Fullerene solar cells. *Phys. Rev. B - Condens. Matter Mater. Phys.* **86**, 1–9 (2012).

32. Vandewal, K. *et al.* Efficient charge generation by relaxed charge-transfer states at organic interfaces. *Nat. Mater.* **13**, 63–68 (2014).

33. Melianas, A. *et al.* Nonequilibrium site distribution governs charge-transfer electroluminescence at disordered organic heterointerfaces. *Proc. Natl. Acad. Sci. U. S. A.* **116**, 23416–23425 (2019).
