# Supplementary material for: Influence of static disorder of charge transfer state on voltage loss in organic photovoltaics
Source: Nat Commun. 2021 Jun 15;12:3642. doi: 10.1038/s41467-021-23975-3 (PMC8206127; doi:10.1038/s41467-021-23975-3)
Supplement: Supplementary file 3 — Solar Cells Reporting Summary [file 41467_2021_23975_MOESM3_ESM.pdf]

nature research

Corresponding author(s): Jun Yan and Jenny Nelson

Solar Cells Reporting Summary

Nature Research wishes to improve the reproducibility of the work that we publish. This form is intended for publication with all accepted papers reporting the characterization of photovoltaic devices and provides structure for consistency and transparency in reporting. Some list items might not apply to an individual manuscript, but all fields must be completed for clarity.

For further information on Nature Research policies, including our data availability policy, see Authors & Referees.

Experimental design

Please check: are the following details reported in the manuscript?

1. Dimensions

Item	Yes	No	Response
Area of the tested solar cells	x		0.045 cm-2
Method used to determine the device area	x		Evaporation mask

2. Current-voltage characterization

Item	Yes	No	Response
Current density-voltage (J-V) plots in both forward and backward direction		x	No relevant to this study
Voltage scan conditions<br>*For instance: scan direction, speed, dwell times*	x		forward scan, 100 mV s-1, 1,000,000 us
Test environment<br>*For instance: characterization temperature, in air or in glove box*	x		300 K, under N2 atmosphere
Protocol for preconditioning of the device before its characterization		x	No specfic preconditioning
Stability of the J-V characteristic<br>*Verified with time evolution of the maximum power point or with the photocurrent at maximum power point; see ref. 7 for details.*		x	Not relevant to this study

3. Hysteresis or any other unusual behaviour

Item	Yes	No	Response
Description of the unusual behaviour observed during the characterization		x	Not relevant to this study
Related experimental data		x	Not relevant to this study

4. Efficiency

Item	Yes	No	Response
External quantum efficiency (EQE) or incident photons to current efficiency (IPCE)	x		See Figure 4
A comparison between the integrated response under the standard reference spectrum and the response measure under the simulator		x	Not relevant to this study
For tandem solar cells, the bias illumination and bias voltage used for each subcell		x	Not relevant to this study

5. Calibration

Item	Yes	No	Response
Light source and reference cell or sensor used for the characterization	x		See Supplementary Method 1
Confirmation that the reference cell was calibrated and certified	x		See Supplementary Method 1

Calculation of spectral mismatch between the reference cell and the devices under test	☐ Yes ☒ No	Not relevant to this study

6. Mask/aperture

Size of the mask/aperture used during testing	☒ Yes ☐ No	0.045 cm-2
Variation of the measured short-circuit current density with the mask/aperture area	☐ Yes ☒ No	Not relevant to this study

7. Performance certification

Identity of the independent certification laboratory that confirmed the photovoltaic performance	☐ Yes ☒ No	Not relevant to this study
A copy of any certificate(s) *Provide in Supplementary Information*	☐ Yes ☒ No	Not relevant to this study

8. Statistics

Number of solar cells tested	☒ Yes ☐ No	32
Statistical analysis of the device performance	☒ Yes ☐ No	All dataare shown with standard derivations

9. Long-term stability analysis

Type of analysis, bias conditions and environmental conditions *For instance: illumination type, temperature, atmosphere humidity, encapsulation method, preconditioning temperature*	☐ Yes ☒ No	Not relevant to this study
